# *Ortho*-functionalization of a ^211^At-labeled aryl compound provides stabilization of the C-At bond against oxidative dehalogenation

**DOI:** 10.1038/s41598-025-01162-4

**Published:** 2025-05-15

**Authors:** Romain Fouinneteau, Clémence Maingueneau, Nicolas Galland, Cécile Perrio, François Guérard

**Affiliations:** 1 Nantes Université, Inserm, CNRS, UMR 1307, CRCI2NA, University of Angers, Nantes, France; 2 CNRS, CEISAM, UMR 6230, Nantes Université, 44000 Nantes, France; 3 UNICAEN, CEA, CNRS, Normandie Univ, Cyceron, Caen, France

**Keywords:** Medicinal chemistry, Nuclear chemistry

## Abstract

Targeted alpha therapy appears to be a promising approach in nuclear medicine for the treatment of cancers. Thanks to its appropriate physical properties, ^211^At is an ideal candidate compared with other alpha emitters. Because of its enigmatic nature, the chemical element astatine is the subject of growing interest to better understand its radiochemistry. The application of ^211^At in the clinic, which has shown good therapeutic results, is however still hampered. Stability issues of ^211^At-radiolabeling were quickly encountered in early preclinical trials and later confirmed in the clinic that mainly studied ^211^At-radiopharmaceuticals labeled by formation of an astatobenzamide derivative. Recent studies have shed light on the deastatination mechanisms encountered in vivo, in particular potential oxidative mechanisms that may weaken the carbon-astatine bond formed during the radiolabeling. In this work, we show that *ortho*-functionalization of astatoaryl compounds with benzyl alcohols protects radiolabeling from deastatination in a strongly oxidizing and acidic medium, as well as in liver microsomal media reproducing in vivo deastatination via cytochrome P450 (CYP450) mediated mechanisms. Our results open the way to the rational design of new ^211^At-aryl-based compounds with improved stability.

**Supplementary Information:**

The online version contains supplementary material available at 10.1038/s41598-025-01162-4.

## Introduction

Astatine-211 is one of the most promising alpha emitters for targeted alpha therapy (TAT) of cancers, which has already shown very encouraging clinical results^1–3^. Unlike other alpha-emitting radioisotopes, such as ^225^Ac, ^223^Ra or ^227^Th, ^211^At is not derived from the purification of nuclear waste, the supply of which is currently a major issue for a sustainable transition to the clinic^4^. It can be produced in a medium-energy cyclotron by bombardment of alpha particles (around 28 MeV) on a ^209^Bi target, a material that is stable, abundant and inexpensive, according to the ^209^Bi(α,2n)^211^At nuclear reaction^5^, making it available worldwide with an appropriate network of cyclotrons. Its physical properties are also ideal, since it emits only a single high-energy alpha particle during its decay unlike above mentioned radionuclides that emit a cascade of alpha and/or beta particles. In addition, its short half-life of 7.2 h reduces issues related to radioactivity released from patients after the cure.

**Fig. 1:**
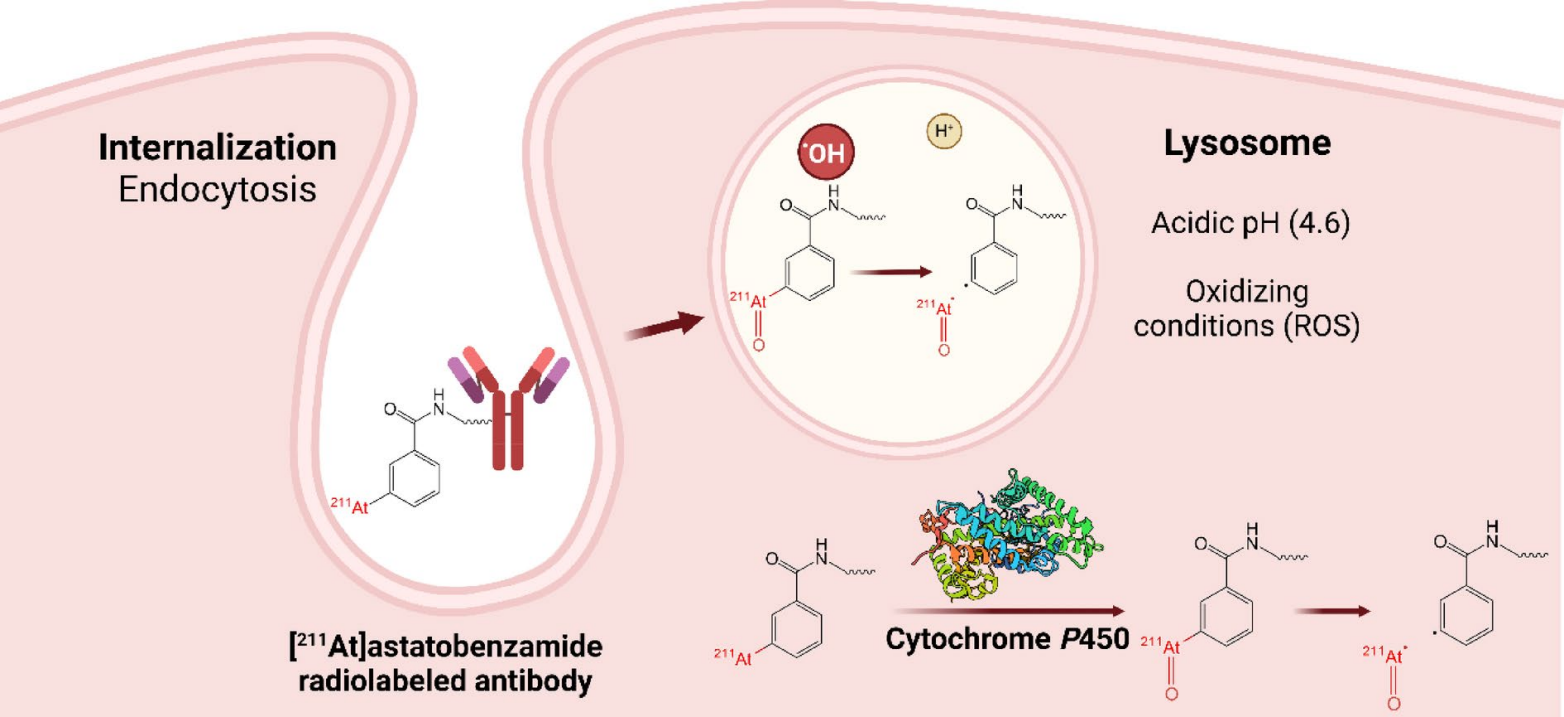
Suggested degradation of the C-At bond after internalization of a radiopharmaceutical in a targeted cell and oxidation in the lysosome environment or by CYP450 enzymes.

**Fig. 2:**
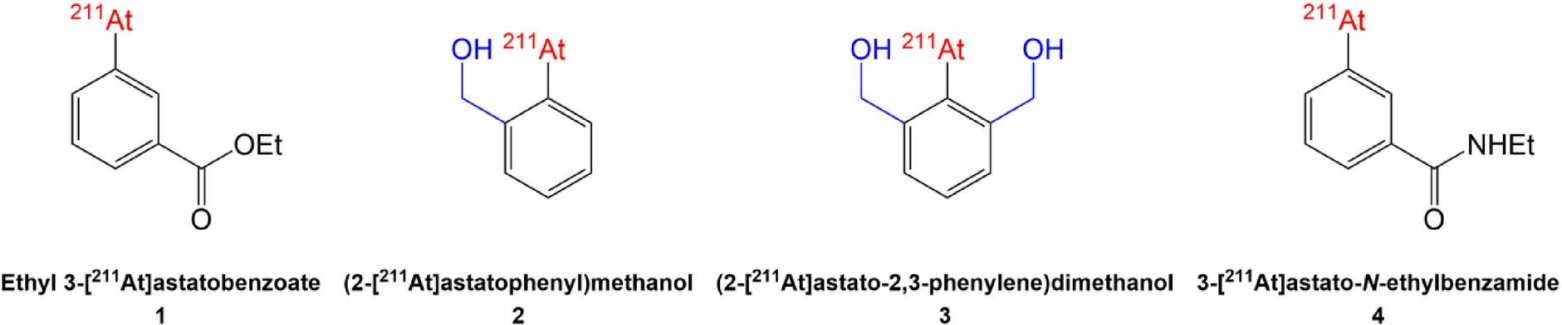
^211^At-compounds discussed in this study.

The main approach to produce ^211^At-radiopharmaceuticals is based on the formation of a covalent bond between astatine and a carbon atom of an aryl moiety. The most relevant example is *N*-succinimidyl-[^211^At]astatobenzoate ([^211^At]SAB), a prosthetic group for bioconjugation to lysine residues of proteins which has been used in a large number of preclinical studies and in the two clinical trials reported to date^6^. Despite promising results, a lack of stability of the ^211^At-labeled compounds is often observed in vivo with this chemical bond, leading to a release of ^211^At. The latter then accumulates in healthy organs such as thyroid, stomach, spleen and lungs, instead of the targeted tumor site, and brings concern as to the safety of use of such radiopharmaceuticals. The in vivo dehalogenation happens especially when carrier molecules are internalized within cells where they may be exposed to an acidic and oxidative environment in lysosomes and to CYP450 redox enzymes largely present in the endoplasmic reticulum (Fig. 1)^7,8^. In addition, the instability is much more pronounced in astatinated compounds than their iodinated analogues^9^. Based on these observations, Teze et al. have investigated the mechanism involved in the in vivo dehalogenation of astatoaryl compounds^10^. A series of assays evidenced an extensive deastatination of an astatobenzoate derivative (Fig. 2 compound **1**), serving as model of [^211^At]SAB, in acidic oxidative media compared to the iodinated analogue that remained intact. These results were complemented by theoretical (DFT) calculations indicating not only that the astatinated compound is significantly easier to oxidize than its iodinated counterpart, but also that for resulting oxidized compounds, the carbon-halogen bond is 25% less stable for At compared to I (bond dissociation energy of 28.2 and 37.8 kcal mol^−1^, respectively). More recently, Li et al. reported the preparation of ^211^At-aryl-based compounds that were oxidized prior to in vivo evaluation. Results clearly indicated a lack of stability of these compounds, reinforcing the hypothesis that oxidation of At is detrimental to the in vivo stability of the labeling^11^.

Building up on these findings, we ambition to design new astatoaryl compounds that would be stable against oxidative deastatination. Herein, we report the investigation of molecules in which the arylastatide is substituted in *ortho* position by one or two benzylic alcohol groups (Fig. 2 compounds **2** and **3**, respectively).

The present study reports the preparation and ^211^At-labeling of corresponding aryliodonium salts precursors followed by the evaluation of the stability of ^211^At-products in a chemical model of acidic/oxidative medium to mimic conditions after cell internalization, especially within lysosomes, and also in liver microsomal medium to reproduce metabolism via CYP450 redox enzymes responsible for dehalogenation and found in tumor cells as well as in the liver. ^211^At-compounds **2** and **3** were compared to 3-[^211^At]astato-*N*-ethylbenzamide ([^211^At]AEB—Fig. 2 compound **4**), a model designed to mimic the astatoaryl moiety found after bioconjugation of [^211^At]SAB to amino groups of proteins.

## Results

**Fig. 3:**
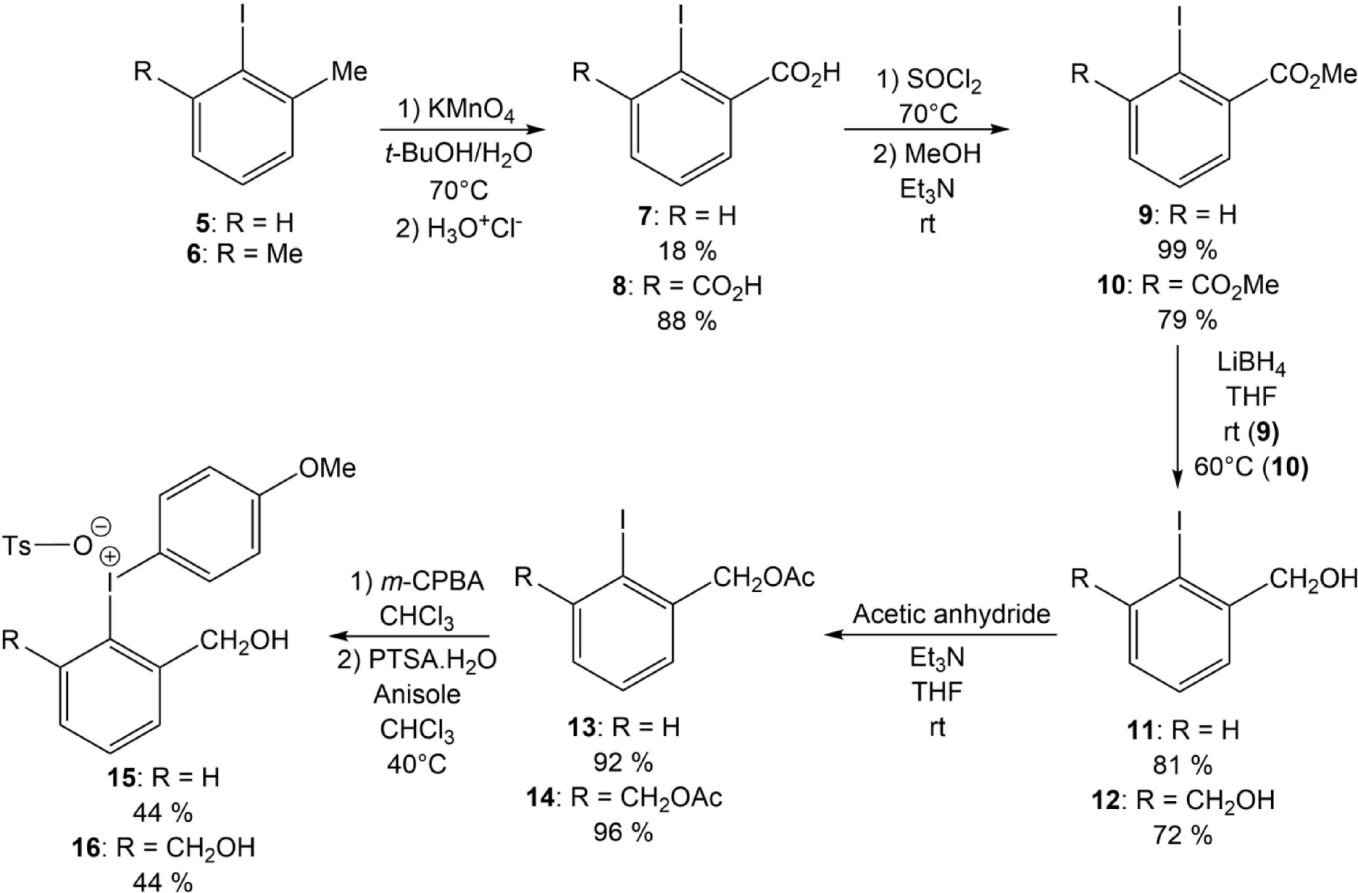
Synthesis of *ortho*-functionalized precursors **15** and **16**.

*Synthesis of precursors.* Recent reports have provided several new ^211^At-labeling approaches, offering an enriched arsenal of strategies that may be selected according the characteristics of the target ^211^At-compound^6^. In our case, nucleophilic ^211^At-labeling of aryliodonium salt precursors appeared as an attractive strategy since it has been reported favorable to produce *ortho*-substituted ^211^At-aryl compounds, even with electron enriched substrates^12^. The synthesis of the iodonium salt precursors of the mono- or di-*ortho*-functionalized compounds **2** and **3** was carried out in five steps from 2-iodotoluene (**5**) and 2-iodo-*m*-xylene (**6**), respectively (Fig. 3). The first step consisted in the oxidation of the methyl group(s) with potassium permanganate which led to the corresponding carboxylic acids **7** and **8**, which were then converted to the methyl esters **9** and **10** via the formation of an acid chloride intermediate. Subsequent reduction with lithium borohydride led to the corresponding benzyl alcohols intermediates **11** and **12**, which also served as chromatographic references to identify the ^211^At-radiolabeled compounds **2** and **3** since no non-radioactive isotopes of astatine exist. Benzyl alcohols were then protected by an acetyl group (**13** and **14**). Finally, iodonium salts were produced following previously reported one-pot two-step procedure: (i) the oxidation of the iodine position with *meta*-chloroperbenzoic acid into the corresponding aryliodane, (ii) followed by a ligand exchange in acid medium with anisole used as low reactive auxiliary ligand to favor nucleophilic substitution on the other aryl group^12^. Interestingly, the formation of iodonium salts **15** and **16** under acidic conditions (presence of tosylic acid) led to the simultaneous deprotection of benzyl alcohols, thus avoiding a deprotection step after radiolabeling. This synthetic route yielded the di-functionalized precursor **16** with an overall yield of 21%, compared with 6% for the mono-functionalized precursor **15**.

**Fig. 4:**
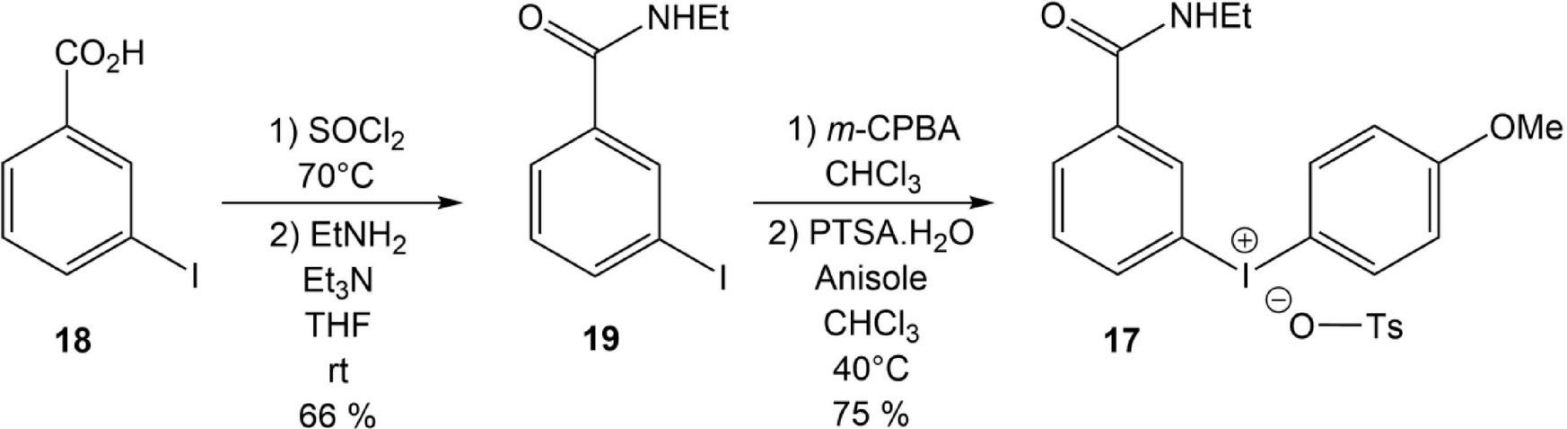
Synthesis of precursor **17**.

^*211*^*At-radiolabeling*. The ^211^At-labeling of aryliodonium salts requires nucleophilic astatine. Accordingly, reduction of astatine to At¯ was performed using dithiotreitol (DTT) following the procedure reported previously^13^. Although acetonitrile is most frequently reported for ^211^At-labeling of aryliodonium salts, methanol was preferred here for precursors solubility reasons. Reactions were then run for 30 min at 60 °C, lower temperatures leading to inconsistent RCYs (Table 1).

Similarly, the precursor (**17**) of [^211^At]AEB model compound, was synthesized in two steps starting from 3-iodobenzoic acid (**18**), which was converted via activation as an acyl chloride to the corresponding ethylbenzamide (**19**), also serving as a chromatographic reference for ^211^At-compound (**4**). The above procedure was then used to produce the aryliodonium salt (**17**) with an overall yield of 50% (Fig. 4).

**Table 1:** Radiolabeling of iodonium salts.


Precursor	RCY I (%)	RCY II (%)
**17**	83 ± 2 (61 ± 27)^a^	9 ± 1 (12 ± 8)^a^
**15**	77 ± 3	4 ± 1
**16**	87 ± 2 (81 ± 10)^a^	1 ± 1 (1 ± 1)^a^

Standard conditions: 95 µL of precursor (2.5 mM in MeOH) and 5 µL of ^211^At- (0.5–100 MBq) reduced with DTT (10 mg mL^−1^) in water (n = 3). RCYs were determined by radio-HPLC analyses of crude product (radio-chromatograms are provided in SI: figures S2, S3 and S4).

^a^Value in parentheses given for reactions run at room temperature.

Despite the presence of free alcohol groups close to the iodine atom in the precursor, high radiochemical yields (RCYs) were obtained, ranging from 77% for the mono-functionalized product **2** to 87% for the di-functionalized product **3**. [^211^At]astatoanisole as potential by-product of aryliodonium salts labeling was observed only in small proportions, with RCYs decreasing from 9 to 1% as the steric hindrance was increased, confirming the favorable impact of *ortho*-substitution previously reported^12^. Each compound was then purified on reverse phase cartridges yielding 31 ± 2% RCY with 98 ± 2% radiochemical purity (RCP), 27 ± 3% RCY with 97 ± 1% RCP, and 27 ± 3% RCY with 98 ± 1% RCP for compounds **4**, **2** and **3**, respectively.

**Fig. 5:**
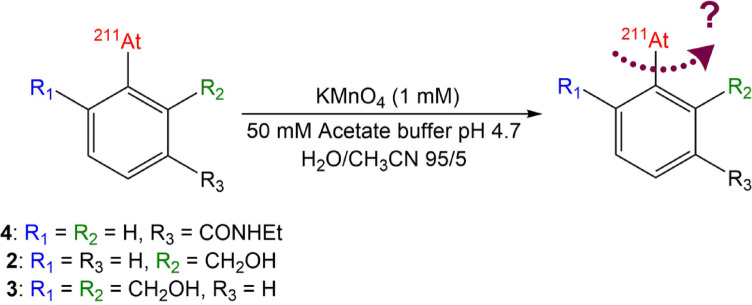
Chemical oxidation assay.

*Stability of *^*211*^*At-labeled compounds in oxidizing media*. The first stability assay aimed at mimicking conditions encountered within lysosomes, i.e., exhibiting both acidic pH and oxidizing potential. Adapted from Teze et al.^10^, it consists in an incubation of the ^211^At-labeled product in 50 mM acetate buffer (pH = 4.7) containing a 1 mM potassium permanganate solution (Fig. 5).

**Fig. 6:**
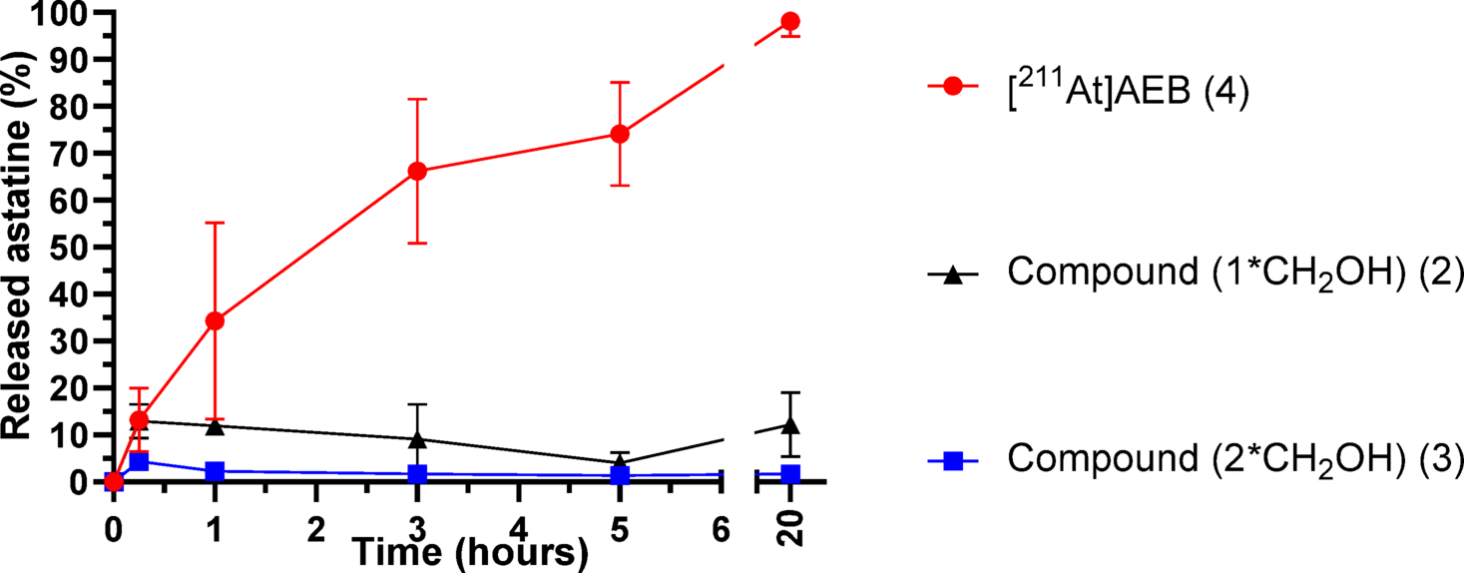
Deastatination of functionalized compounds 2 and 3 in oxidizing medium compared to [^211^At]AEB (**4**) (n = 3).

HPLC analyses of the oxidizing medium revealed almost complete degradation of the [^211^At]AEB after 20 h of incubation, with free astatine detected as the major radioactive signal (radio-chromatograms provided in Figure S5). These results are consistent with those of Teze et al. using a very similar compound, ethyl 3-[^211^At]astatobenzoate **1**, which showed rapid degradation under similar conditions^10^. For the dihydroxy compound **3**, a completely different result was obtained, with virtually no free astatine observed after 20 h incubation (≤ 1%). However, the starting material was progressively converted to a more polar species (Figure S6). It is known that permanganate can oxidize easily benzyl alcohol to carboxylic acids. Accordingly, 2-iodo-isophtalic acid (**8**) was injected into the HPLC system (Figure S6) and showed a retention time similar to the degradation product, strongly suggesting the formation of 2-[^211^At]astato-isophtalic acid (**20**). A similar result was obtained with the mono-functionalized compound **2**, with a significantly reduced release of astatine after 20 h of incubation compared to reference compound **4**, which reached 12%. These results highlight the stabilizing effect of benzyl alcohols on oxidation, with a progressive effect on stability in an oxidizing environment depending on the number of benzyl alcohols present in *ortho* position of astatine (Fig. 6).

**Fig. 7:**
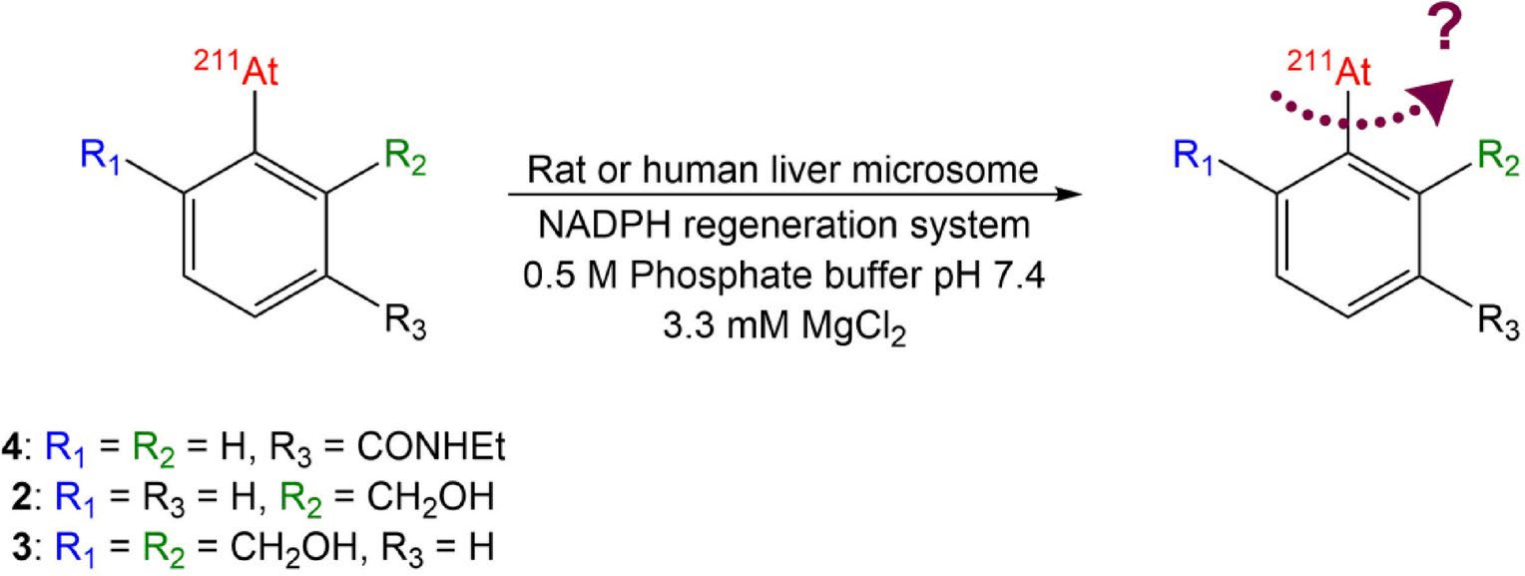
Conditions used for liver microsome stability assays.

Since oxidation by CYP450 enzymes is a plausible cause of oxidative deastatination within targeted cells or in the liver, we also assessed the stability of our compounds against liver microsomes. Liver microsomes contain CYP450, a family of oxidase enzymes that were used in our assay, adapted from Schmitt et al.^14^ and using a nicotinamide adenine dinucleotide phosphate (NADPH) redox regeneration system to regenerate the redox properties of CYP450. For comparison, we performed the stability assay in rat and human liver microsomes (Fig. 7).

**Fig. 8:**
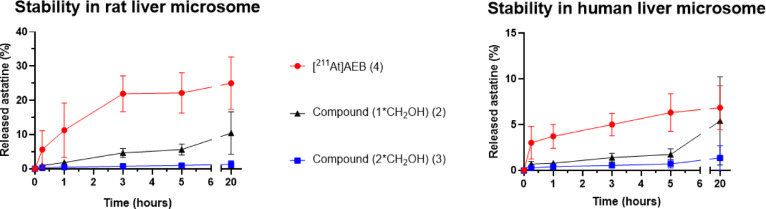
Monitoring of deastatination of functionalized compounds in microsomal medium compared to [^211^At]AEB (n = 3).

HPLC analyses of the stability medium show the same trend than in the permanganate assay, with [^211^At]AEB (**4**) showing a rapid deastatination, with 25% and 6% of free astatine measured after 20 h incubation in rat and human microsomes, respectively. The rest of the activity corresponds to the formation of more polar metabolites, which was attributed to the CYP450 oxidative activity at the amide bond, and practically no presence of the starting compound after 20 h incubation (Figures S7-S8). In contrast, the difunctionalized compound (**3**) appeared very stable, with practically no release of astatine in rat or human microsomes (< 2% for both). No formation of metabolites was observed, the product remaining intact probably because of the absence of oxidation sites for the CYP450 enzymes (Figures S9-S10). An intermediate result was obtained for the monofunctionalized compound (**2**), with an astatine release of around 10% and 5% in rat and human microsomes, respectively. These results once again support the gradual effect of the number of benzyl alcohols on stability (Fig. 8). With all three compounds, dehalogenation was more rapid in rat than in human microsomes.

A noticeable difference between our simplified model compounds and [^211^At]AEB is the presence of an amide group. To ensure that the release of At in our assays was not due to this structural difference, we also performed a rat liver microsome stability assay on [^211^At]astatobenzene, which showed a similar profile to the result obtained with [^211^At]AEB in the same medium (Figure S11). This result therefore rules out a possible destabilizing effect of the amide function compared with *ortho*-functionalized compounds which do not possess one.

## Discussion

The stabilization of the ^211^At-labeled for radiopharmaceutical candidates is still a major challenge in order to provide the safest use of this radionuclide of high interest. After an era of radiolabeling strategies solely based on the formation of an At-aryl bond, alternatives were proposed with various degrees of success: the bonding of astatine to a boron atom was investigated with boron clusters that proved successful^15^, while binding to a metallic center such as Rh/Ir(III)^16^ or Rh(I)^17^ is promising, and more recently the alkyl-At bond was shown to be also usable^18^. Yet, the possibility of diversification around the phenyl moiety together with the large presence of (hetero)aromatic patterns in small to large biomolecule of interest for cancer targeting, make the optimization of the astatoaryl group a relevant strategy to investigate. To date a limited number of variations around the SAB structure, the current gold standard that however exhibits insufficient in vivo stability, were reported. Only one has shown evidence of improved in vivo stability, *N*-succinimidyl 3-[^211^At]astato-4-guanidinomethylbenzoate (SAGMB) and its positional isomer *iso*-SAGMB^19,20^. The improved stability of this compounds was recently attributed to the guanidinium substituent that is able to prevent the astatine atom to form halogen-bond interactions with the selenocysteine residue of type 1 or type 3 iodothyronine deiodinases, leading to a C-At bond breakage, similarly to the known mechanism of in vivo deiodination of iodoaryl compounds^21^. Another deastatination mechanism was reported, Teze et al. attributing the higher C-At bond degradation over C-I bond to oxidative and acidic conditions found within lysosomes of the targeted cells^10^. Accordingly, we have studied the possibility to produce astatoaryl compounds that would be stable against oxidative dehalogenation.

The preparation of two new *ortho*-functionalized astatoaryl compounds with benzyl alcohols was successfully achieved using the iodonium salt radiolabeling method. To assess the stability of these compounds, two stability assays were carried out, either in strongly oxidizing medium provided by permanganate, or in liver microsomes for exposure of compounds to oxidative CYP450 enzymes. In both assays, *ortho* functionalized compounds **2** and **3** exhibited improved stability against deastatination compared to the reference compound [^211^At]AEB (**4**). In addition, degradation rate was gradually decreased when *ortho* functionalization was increased from 0 to 2. Interestingly, microsomal media assays indicated no alteration of starting material (**3**), while permanganate assay indicated some evolution of the compound which was attributed to the oxidation of the benzyl alcohol to carboxylic acid, but the At atom did remain firmly bound to its carbon atom. The steric hindrance and the hydrophilicity provided by the alcohol groups in the At surrounding may in part explain the reduced degradation by disturbing CYP450 enzymes approach on the halogen site. However, these arguments are not sufficient for explaining the stability in the purely chemical oxidation assay found in permanganate medium. Similarly to aryliodinanes formed by oxidation of *ortho*-functionalized aryliodides as previously reported by Amey and Martin^22^, stabilization by the *ortho* alcohol arms through cyclization into an arylakoxyastatinane, could also be advanced. We were however unable to confirm this hypothesis by experimental observation, only conversion of benzylic alcohol into carboxylic acids being detected. Such compounds, if formed, may actually exist only transiently making their detection difficult.

Taken together, these results show that *ortho*-functionalization by benzylic alcohol groups is a promising strategy to protect astatoaryl compounds from oxidative degradation that we simulated in vitro. Our results are in line with the work of Hirata et al. published during revision of this article which evidenced an improved in vivo stability of a similar astatobenzene compound also substituted by benzyl alcohol groups in *ortho* positions^23^. In their study, the authors investigated an analogue of our compound **3**, differing by the presence of a carboxylic acid in *para* position relative to astatine and conjugated to glycine, resulting in an hyppuric acid derivative. They evaluated it in a biodistribution study in normal mice, the results of which indicating a decrease in stomach and spleen uptake by approximately 50% compared to the analogue devoid of hydroxyl groups, which is indicative of an increased in vivo stability of the C-At bond and confirms the potential of *ortho*-substitution to stabilize astatoaryl based radiopharmaceuticals. In addition to the protection against oxidation discussed herein, it can be envisaged that a protection against halogen-bond mediated mechanisms of dehalogenation would be provided by the hydroxyl groups according to the recent report by Yssartier et al.^21^ Their propensity to form hydrogen bonds could offset astatine’s ability to interact with the biological environment through halogen bonds. Moreover, the addition of benzyl alcohol makes the molecule more polar, hypothetically affecting less the biodistribution of small vectors such as peptides than most previously reported At-aryl-based labeling strategies. Although the scope of applicability of this stabilization strategy remains to be confirmed by the study of representative (hetero)aryl compounds diversely substituted at the *meta* and *para* positions, recent works by the Uehara group support the potential of stabilization of hydroxyl groups in a different C-At bond environment, as illustrated by the development of the in vivo stable neopentyl glycol group comprised of an alkyl-astatine bond^24,25^. In view of our results, development of functionalized derivatives for conjugation to biologically relevant molecules as well as other *ortho* substituents is in progress and their in vivo evaluation will be reported in due time.

## Methods

### Synthesis

#### Material and instrumentation

Commercial reagents and solvents were purchased from Merck, Fisher Scientific, TCI or Fluorochem. Deuterated solvents were provided from Merck. Reactions were monitored by thin-layer chromatography (TLC) using 60 F254 silica gel plates on aluminium support (Merck) and revealed by UV lamp (254 nm). A Puriflash 430 (Interchim) with 30 μm or 15 μm silica pre-packed columns was used to purify the compounds. NMR analyses were performed with a Bruker AC spectrometer operating at 400 MHz (^1^H) or 101 MHz (^13^C). Chemical shifts (δ) are expressed in part per million (ppm) relative to deuterated solvents, CDCl_3_: 7.26 (^1^H), 77.16 (^13^C) ppm, DMSO-d_6_: 2.50 (^1^H), 39.52 (^13^C) ppm, MeOD: 3.31 (^1^H), 49.00 (^13^C) ppm. The multiplicity is reported with the following symbols: s (singlet), d (doublet), t (triplet), q (quadruplet) and m (multiplet). Mass spectrometry analyses were operated on a Synapt G2 HRMS Q-TOF mass spectrometer (Waters) equipped with an electrospray ionization (ESI) interface operating in the positive mode.

*2-Iodo-benzoic acid (7)*. A *tert*-BuOH/water (60 mL/40 mL) mixture was added to a flask containing the commercially available starting material 1-iodo-2-methylbenzene **5** (5.00 g, 1 eq.). A first portion of KMnO_4_ (4.50 g, 1.25 eq.) was added to the medium and stirred vigorously at room temperature. The medium was stirred for 2 h at 70 °C and then, after cooling to room temperature, the second portion of KMnO_4_ (4.50 g, 1.25 eq.) was added. The medium was stirred at 70 °C overnight. The medium was hot-filtered over fritted funnel and the solid rinsed with water. The filtrate was acidified to pH 2 with 37% hydrochloric acid. The product was extracted with 3 × 35 mL of ethyl acetate, then the combined organic layers were dried over anhydrous magnesium sulfate, filtered and concentrated to give product **7** as a brown solid (1.05 g, 18%).

^1^H NMR (400 MHz, DMSO-d_6_): δ (ppm) 7.98 (dd, *J* = 7.8, 1.1 Hz, 1H), 7.71 (dd, *J* = 7.7, 1.7 Hz, 1H), 7.48 (td, *J* = 7.6, 1.2 Hz, 1H), 7.23 (td, *J* = 7.7, 1.7 Hz, 1H).

^13^C NMR (101 MHz, DMSO-d_6_): δ (ppm) 168.1, 140.5, 136.9, 132.4, 130.0, 128.1, 94.0.

*2-Iodo-isophtalic acid (8)*. A *tert*-BuOH/water (50 mL/40 mL) mixture was added to a flask containing the commercially available 2-iodo-m-xylene **6** (3.00 g, 1 eq.). A first portion of KMnO_4_ (5.10 g, 2.5 eq.) was added to the solution and stirred vigorously at room temperature. The medium was stirred for 2 h at 70 °C and, after cooling to room temperature, the second portion of KMnO_4_ (5.10 g, 2.5 eq.) was added. The medium was stirred at 70 °C overnight. The medium was then hot-filtered over a frit funnel and the solid rinsed with water. The filtrate was acidified to pH 2 with 37% hydrochloric acid. The product was extracted with 3 × 25 mL of ethyl acetate, then the combined organic layers were dried over anhydrous magnesium sulfate, filtered and concentrated to dryness to give product **8** as a white solid (3.32 g, 88%).

^1^H NMR (400 MHz, DMSO-d_6_): δ (ppm) 13.50 (s, 2H), 7.60–7.55 (m, 2H), 7.51 (dd, *J* = 8.6, 6.3 Hz, 1H).

^13^C NMR (101 MHz, DMSO-d_6_): δ (ppm) 169.2, 141.2, 129.6, 128.2, 91.0.

*2-Iodo-benzoic acid methyl ester (9)*. Thionyl chloride (10 mL) was added to a flask containing the carboxylic acid derivative **7** (1.00 g, 1 eq.) under an inert atmosphere. The mixture was stirred at 70 °C during 4 h. Excess thionyl chloride was removed by distillation under reduced pressure. The residue was then placed in an ice bath and methanol (10 mL) and triethylamine (5 mL) were added slowly, then stirred at room temperature for 2 days. The reaction was quenched with water and the product extracted with 2 × 15 mL of dichloromethane. Organic layer was washed with 20 mL of water and 20 mL of brine and dried over anhydrous magnesium sulfate. After filtration and solvent evaporation, the product was purified by flash chromatography on a silica cartridge (eluent: gradient of dichloromethane/methanol) to give product **9** as a colorless oil (1.05 g, 99%).

^1^H NMR (400 MHz, MeOD): δ (ppm) 8.01 (dd, *J* = 8.0, 1.2 Hz, 1H), 7.74 (ddd, *J* = 7.8, 3.0, 1.8 Hz, 1H), 7.46 (ddt, *J* = 8.8, 7.7, 1.1 Hz, 1H), 7.26–7.19 (m, 1H), 3.91 (d, *J* = 0.9 Hz, 3H).

^13^C NMR (101 MHz, CDCl_3_): δ (ppm) 167.1, 141.5, 135.3, 132.8, 131.1, 128.0, 94.2, 52.6.

*2-Iodo-isophtalic acid dimethyl ester (10)*. Thionyl chloride (5 mL) was added to a flask containing the carboxylic acid derivative **8** (570 mg, 1 eq.) under an inert atmosphere. The mixture was stirred at 70 °C overnight. Excess thionyl chloride was removed by distillation under reduced pressure. The residue was then placed in an ice bath and methanol (10 mL) and triethylamine (2 mL) were added slowly, then stirred at room temperature overnight. The reaction was quenched with water, and the product extracted with dichloromethane. The organic layer was dried over anhydrous magnesium sulfate, filtered and evaporated. The residue was purified by flash chromatography on a silica cartridge (eluent: gradient of chloroform/methanol) to give product **10** as a yellow oil (490 mg, 79%).

^1^H NMR (400 MHz, MeOD): δ (ppm) 7.62–7.58 (m, 2H), 7.53–7.46 (m, 1H), 3.92 (s, 6H).

^13^C NMR (101 MHz, MeOD): δ (ppm) 169.8, 141.6, 131.9, 129.3, 91.6, 53.2.

*(2-Iodo-phenyl)-methanol (11)*. A solution of methyl ester **9** (800 mg, 1 eq.) in anhydrous THF (15 mL) was placed in a flask under inert atmosphere. A solution of LiBH_4_ (100 mg in 5 mL THF, 1.5 eq.) was added dropwise at 0 °C with vigorous stirring. The mixture was then stirred at rt for one day then placed in an ice bath, and the reaction quenched by gently adding 0.4 M HCl to pH 6–7. After evaporation of the solvent, the residue was purified by flash chromatography on a silica cartridge (eluent: gradient of chloroform/methanol) to give product **11** as a white solid (580 mg, 81%).

^1^H NMR (400 MHz, DMSO-d_6_): δ (ppm) 7.80 (dd, *J* = 7.9, 1.2 Hz, 1H), 7.51–7.45 (m, 1H), 7.41 (td, *J* = 7.4, 1.2 Hz, 1H), 7.02 (td, *J* = 7.5, 1.8 Hz, 1H), 5.48 (t, *J* = 5.5 Hz, 1H), 4.40 (d, *J* = 5.0 Hz, 2H).

^13^C NMR (101 MHz, DMSO-d_6_): δ (ppm) 143.7, 138.4, 128.8, 128.2, 127.6, 96.9, 67.3.

*(3-Hydroxymethyl-2-iodo-phenyl)-methanol (12)*. A solution of methyl ester **10** (1.02 g, 1 eq.) in anhydrous THF (19 mL) was placed in a flask under inert atmosphere. A solution of LiBH_4_ (2 M in THF, 3 eq.) was added dropwise at 0 °C with vigorous stirring. The mixture was stirred at 60 °C for one night then placed in an ice bath. The reaction quenched by gently adding 1 M HCl until end of gas evolution, then the solvent was evaporated. The residue was purified by flash chromatography on a silica cartridge (eluent: gradient of dichloromethane/methanol) to give product **12** as a white solid (606 mg, 72%).

^1^H NMR (400 MHz, MeOD): δ (ppm) 7.44–7.31 (m, 3H), 4.62 (s, 4H).

^13^C NMR (101 MHz, MeOD): δ (ppm) 144.8, 129.2, 127.7, 100.3, 70.0.

*Acetic acid 2-iodo-benzyl ester (13)*. The alcohol **11** (580 mg, 1 eq.) was dissolved in anhydrous THF (20 mL) in a dry flask under an inert atmosphere. The resulting solution was placed at 0 °C, then triethylamine (0.52 mL, 1.5 eq.) and acetic anhydride (0.30 mL, 1.3 eq.) were added and stirred at room temperature for 4 days. After evaporation of the solvent, the obtained solid was dissolved in dichloromethane. The organic layer was washed with 2 × 20 mL of water, dried over MgSO_4_, filtered and concentrated. The residue was purified by flash chromatography on a silica cartridge (eluent: gradient of dichloromethane/heptane) to give the product **13** as a colorless oil (0.63 g, 92%).

^1^H NMR (400 MHz, CDCl_3_): δ (ppm) 7.86 (dd, *J* = 7.9, 1.1 Hz, 1H), 7.44–7.30 (m, 2H), 7.03 (ddd, *J* = 7.8, 6.7, 2.4 Hz, 1H), 5.12 (s, 2H), 2.15 (s, 3H).

^13^C NMR (101 MHz, CDCl_3_): δ (ppm) 170.7, 139.7, 138.5, 130.0, 129.6, 128.5, 98.5, 70.2, 21.0.

*Acetic acid 3-acetoxymethyl-2-iodo-benzyl ester (14)*. The alcohol **12** (100 mg, 1 eq.) was dissolved in anhydrous THF (5 mL) in a dry flask under an inert atmosphere. The medium resulting solution was placed at 0 °C, then triethylamine (170 µL, 3 eq.) and acetic anhydride (93 µL, 2.5 eq.) were added and stirred at room temperature for 5 days. After evaporation of the solvent, the obtained solid was taken up in dichloromethane. The organic layer was washed with 10 mL of water and 10 mL of brine. The aqueous layer was extracted with 15 mL dichloromethane. The combined organic layers were dried over MgSO_4_, filtered and the solvent was evaporated to give product **14** as a yellow oil which crystallizes at room temperature (127 mg, 96%).

^1^H NMR (400 MHz, CDCl_3_): δ (ppm) 7.37–7.30 (m, 3H), 5.17 (s, 4H), 2.15 (s, 6H).

^13^C NMR (101 MHz, CDCl_3_): δ (ppm) 170.6, 139.5, 129.1, 128.5, 102.7, 70.8, 21.0.

*((2-hydroxymethyl)phenyl)(4-methoxyphenyl)iodonium 4-methylbenzenesulfonate (15)*. The protected alcohol **13** (300 mg, 1 eq.) was dissolved in dichloromethane (10 mL) in a dry flask under inert atmosphere and the *m*-CPBA (230 mg, 1.2 eq.) was added. The solution was stirred at room temperature for 15–20 min, then PTSA.H_2_O (250 mg, 1.2 eq.) and anisole (640 µL, 5.4 eq.) were added. Themixture was heated to 40 °C for 2.5 h. The solvent was evaporated and the residue was purified by flash chromatography on silica cartridges (eluent: gradient of dichloromethane/methanol) to give the iodonium salt precursor **15** as a brown solid (247 mg, 44%).

^1^H NMR (400 MHz, MeOD): δ (ppm) 8.12–8.03 (m, 2H), 7.71–7.65 (m, 2H), 7.58–7.45 (m, 2H), 7.32 (dd, *J* = 5.5, 1.9 Hz, 2H), 7.21 (d, *J* = 7.9 Hz, 2H), 7.18–7.10 (m, 2H), 4.92 (s, 2H), 3.90 (s, 3H), 2.36 (s, 3H).

^13^C NMR (101 MHz, MeOD): δ (ppm) 165.0, 142.2, 141.6, 140.3, 132.6, 132.5, 132.1, 131.0, 129.8, 127.0, 119.2, 114.5, 101.2, 65.5, 56.4, 21.3.

HRMS: C_14_H_14_O_2_I^+^calc: 341.0038, found: 341.0041.

*(2,6-Bis-hydroxymethyl-phenyl)-(4-methoxy-phenyl)-iodonium 4-methylbenzenesulfonate (16)*. The protected alcohol **14** (166 mg, 1 eq.) dissolved in chloroform (15 mL) was placed in a dry flask under inert atmosphere, and the *m*-CPBA (99 mg, 1.2 eq.) was added. The solution was stirred at room temperature for 15–20 min, then PTSA.H_2_O (109 mg, 1.2 eq.) and anisole (280 µL, 5.4 eq.) were added. The mixture was heated to 40 °C for 2.5 h and the solvent was evaporated. The resulting solid was dissolved in the minimum amount of methanol, then diethyl ether was added until a cloud forms and placed in the fridge for one night. The liquid was separated from the brown aggregate formed, which was redissolved in the minimal volume of methanol. Diethylether was added until the solution became cloudy, then the flask was placed in the fridge until crystals appear. The crystals were filtered and rinsed with a cold diethyl ether, then dried in vacuo to give the iodonium salt precursor **16** as brown crystals (114 mg, 44%).

^1^H NMR (400 MHz, MeOD): δ (ppm) 8.09–8.05 (m, 2H), 7.71–7.67 (m, 2H), 7.70–7.62 (m, 1H), 7.59 (d, *J* = 8.2 Hz, 2H), 7.23 (d, *J* = 8.2 Hz, 2H), 7.05–7.01 (m, 2H), 4.80 (s, 4H), 3.83 (s, 3H), 2.37 (s, 3H).

^13^C NMR (101 MHz, MeOD): δ (ppm) 164.3, 145.7, 143.6, 141.6, 138.4, 134.4, 131.2, 129.8, 127.0, 120.3, 118.6, 102.8, 67.2, 56.3, 21.3.

HRMS: C_15_H_16_O_3_I^+^calc: 371.0144, found: 371.0144.

*3-Iodo-N-ethylbenzamide (19)*. Thionyl chloride (5 mL) was added to a dry, inert flask containing commercially available 3-iodobenzoic acid **18** (2.00 g, 1 eq.). The medium was heated under reflux and stirred for 3 h. Excess thionyl chloride was evaporated and anhydrous THF (10 mL) was added to the acid chloride. The mixture was placed in an ice bath and triethylamine (1.36 mL, 1.2 eq.) followed by ethylamine (2 M solution in THF, 4.80 mL, 1.2 eq.) were added slowly. The mixture was left to stir at room temperature under argon for approximately 16 h. The solvent was evaporated, the resulting solid dissolved in dichloromethane. The organic layer was washed with 25 mL of 2 M HCl, 25 mL of saturated Na_2_CO_3_ and finally 25 mL of brine then dried over MgSO_4_, filtered and concentrated. The residue was purified by flash chromatography on a silica cartridge (Eluent: gradient of dichloromethane/AcOEt) to give product **19** as a brown-orange solid (1.46 g, 66%).

^1^H NMR (400 MHz, CDCl_3_): δ (ppm) 8.08 (t, *J* = 1.7 Hz, 1H), 7.81 (dd, *J* = 8.0, 1.4 Hz, 1H), 7.71 (dt, *J* = 7.8, 1.4 Hz, 1H), 7.16 (td, *J* = 7.8, 1.4 Hz, 1H), 6.12 (s, 1H), 3.54–3.43 (m, 2H), 1.25 (t, *J* = 7.3 Hz, 3H).

^13^C NMR (101 MHz, MeOD): δ (ppm) 168.3, 141.5, 137.9, 137.3, 131.3, 127.5, 94.7, 35.9, 14.8.

*((3-Ethylcarbamoyl)phenyl)(4-methoxy-phenyl)iodonium 4-methylbenzenesulfonate (17)*. Compound **19** (600 mg, 1 eq.) was dissolved in dichloromethane (20 mL) in a dry flask under an inert atmosphere and m-CPBA (450 mg, 1.2 eq.) was added. The solution was stirred at room temperature for 15–20 min, then PTSA.H_2_O (500 mg, 1.2 eq.) and anisole (1.28 mL, 5.4 eq.) were added. The mixture was heated to 40 °C for 2 h. The solvent was evaporated and the resulting solid was dissolved in the minimum of methanol. The partially insoluble white solid corresponding to the product was filtered and recovered. The filtrate was evaporated and the operation was repeated until complete recovery of the product. When all the white solid was recovered, the solids of each extraction were combined and dissolved in methanol then crystallized with diethylether to give the precursor **17** as white crystals (903 mg, 75%).

^1^H NMR (400 MHz, MeOD): δ (ppm) 8.54 (t, *J* = 1.8 Hz, 1H), 8.25 (ddd, *J* = 8.1, 2.0, 1.0 Hz, 1H), 8.15–8.07 (m, 2H), 8.06 (dt, *J* = 7.8, 1.3 Hz, 1H), 7.73–7.66 (m, 2H), 7.60 (t, *J* = 7.9 Hz, 1H), 7.26–7.20 (m, 2H), 7.11–7.03 (m, 2H), 3.85 (s, 3H), 3.46–3.33 (m, 2H), 2.37 (s, 3H), 1.22 (t, *J* = 7.3 Hz, 3H).

^13^C NMR (101 MHz, MeOD): δ (ppm) 167.0, 164.7, 143.6, 141.6, 139.3, 138.7, 138.4, 135.0, 133.0, 131.7, 129.8, 127.0, 119.0, 116.6, 104.5, 56.4, 36.1, 21.3, 14.7.

HRMS: C_16_H_17_NO_2_I^+^calc: 382.0304, found: 382.0302.

### Radiochemistry

^211^At was produced at the Arronax cyclotron facility using the ^209^Bi(α,2n) ^211^At reaction and recovered from the irradiated target in chloroform using a dry distillation protocol adapted from the procedure previously reported by Lindegren et al.^26^ Before use, the ^211^At solution was reduced to dryness under a gentle stream of nitrogen. HPLC analyses were performed on a Waters Alliance e2695 system equipped with a Beta-RAM Radio Flow Detector (LabLogic) and a C18 column (Spherisorb ODS2 5l 4.6 mm 25 cm, Waters) with the flow rate set at 1.20 mL/min with the following gradient: t = 0: 90% A, 10% B; t = 7 min: 30% A, 70% B; t = 11–15 min: 100% B with A = H_2_O with 0.05% TFA and B = CH_3_CN with 0.05% TFA. To quantify free astatine that remains trapped in the HPLC system during the elution, 50 µL of an aqueous sodium sulfite solution (10 mg mL^−1^) were injected after each analysis to release free astatine from the HPLC system with the flow rate set at 1.20 mL/min with the following gradient: t = 0: 90% A, 10% B; t = 2–6 min: 100% B with A = H_2_O with 0.05% TFA and B = CH_3_CN with 0.05% TFA. The non-radioactive iodinated compounds were analyzed using this HPLC system and their retention times were used as references for identification of their astatinated analogues. The radio-chromatogram analysis procedure is provided in SI.

#### General procedure for astatination of aryliodonium salts precursors

The dried ^211^At (0.5–120 MBq) was reduced to its nucleophilic form by the addition of 5 µL of DTT (10 mg mL^−1^ in water). After stirring for 1 min at room temperature, 95 µL of a solution of the precursor in methanol (2.5 mM) was added and the medium was placed at 60 °C for 30 min. After HPLC analysis of an aliquot of the medium, it was diluted in 1 mL of water and deposited on a Sep-Pak Plus Long C18 column (Waters) previously conditioned by passing 10 mL of CH_3_CN and 10 mL of water. Excess precursor was removed by passing 3 mL of 95/5 H_2_O/CH_3_CN, then the radiolabeled compound was recovered by passing 1 mL fractions of 1:1 H_2_O/CH_3_CN. The fractions containing the pure radiolabeled compound were combined and diluted in 20 mL of water, and deposited on a Sep-Pak Plus Light C18 column (Waters) previously conditioned by passing 5 mL of CH_3_CN and 5 mL of water. After deposition, the column was air-dried and the pure radiolabeled compound was eluted by passing 500 µL of CH_3_CN. The acetonitrile was evaporated to about half of its initial volume under a stream of dry nitrogen before performing the stability assays.

### Stability assays in oxidizing/acidic medium

A vial was filled with 918 µL of 50 mM acetate buffer (pH 4.7) and 32 µL of 31.6 mM KMnO_4_ solution. After gentle shaking, 50 µL of radiolabeled compound was added to the medium, which was left to incubate at room temperature under vigorous agitation. The medium was analyzed by HPLC after 15 min, 1 h, 3 h, 5 h and 20 h, by withdrawing a small quantity of the medium (5 µL–20 µL) diluted in water into an HPLC vial.

### Stability assay in microsomal medium (rat and human)

In a vial containing 200 µL of 0.5 M phosphate buffer (pH 7.4) and 3.3 mM MgCl_2_, were added 50 µL of NADPH regeneration system A and 10 µL of system B (NADPH system from Promega®). 40 µL (2 × 20 µL with stirring for 30 s between each addition) of radiolabeled compound in CH_3_CN and 675 µL of purified water were added to the medium. The medium was pre-incubated for 5 min at 37 °C. Liver microsomes (rat (Sprague–Dawley, male obtained commercially from Sigma-Aldrich) or human from Sigma-Aldrich) were then added (25 µL–10 mg/mL protein) and the medium was incubated at 37 °C for 20 h. 100 µL of medium were sampled after 15 min, 1 h, 3 h, 5 h and 20 h and the enzymatic reaction rapidly quenched by adding 200 µL of ice-cold MeOH. The sample was diluted with 600 µL of water and centrifuged (4000 rpm, 5 min). The supernatant then was analyzed by HPLC.

## Electronic supplementary material

Below is the link to the electronic supplementary material.

Supplementary Material 1

## Data Availability

All data generated or analyzed during this study are included in this published article (and its Supplementary Information files).
