# Supplementary material for: Ortho-functionalization of a 211At-labeled aryl compound provides stabilization of the C-At bond against oxidative dehalogenation
Source: Sci Rep. 2025 May 15;15:16877. doi: 10.1038/s41598-025-01162-4 (PMC12081905; doi:10.1038/s41598-025-01162-4)
Supplement: Supplementary file 1 — Supplementary Material 1 [file 41598_2025_1162_MOESM1_ESM.docx]

# *Supplementary information: Ortho*-functionalization of a ^211^At-labeled aryl compound provides stabilization of the C-At bond against oxidative dehalogenation

Romain Fouinneteau^a^, Clémence Maingueneau^a^, Nicolas Galland^b^, Cécile Perrio^c*^, François Guérard^a*^

^a^ Nantes Université, Inserm, CNRS, Université d’Angers, UMR 1307, CRCI2NA Nantes, France

^b^ Nantes Université, CNRS, CEISAM, UMR 6230, F-44000 Nantes, France

^c^ Normandie Univ, UNICAEN, CEA, CNRS, Cyceron, Caen, France

*Corresponding author: francois.guerard@univ-nantes.fr, perrio@cyceron.fr

## I) Radio-chromatogram analysis procedure

Analysis of a stability medium is a two-stage process (Figure S1):

1. Injection of an aliquot of the medium into the HPLC. The injection volume is pre-defined according to the volume activity of the medium analyzed (injection of approximately 10 kBq).
2. Following analysis of the medium, a sodium sulfite solution (50µL - 10mg/mL) is injected to wash the HPLC system of any free astatine remaining on the column.


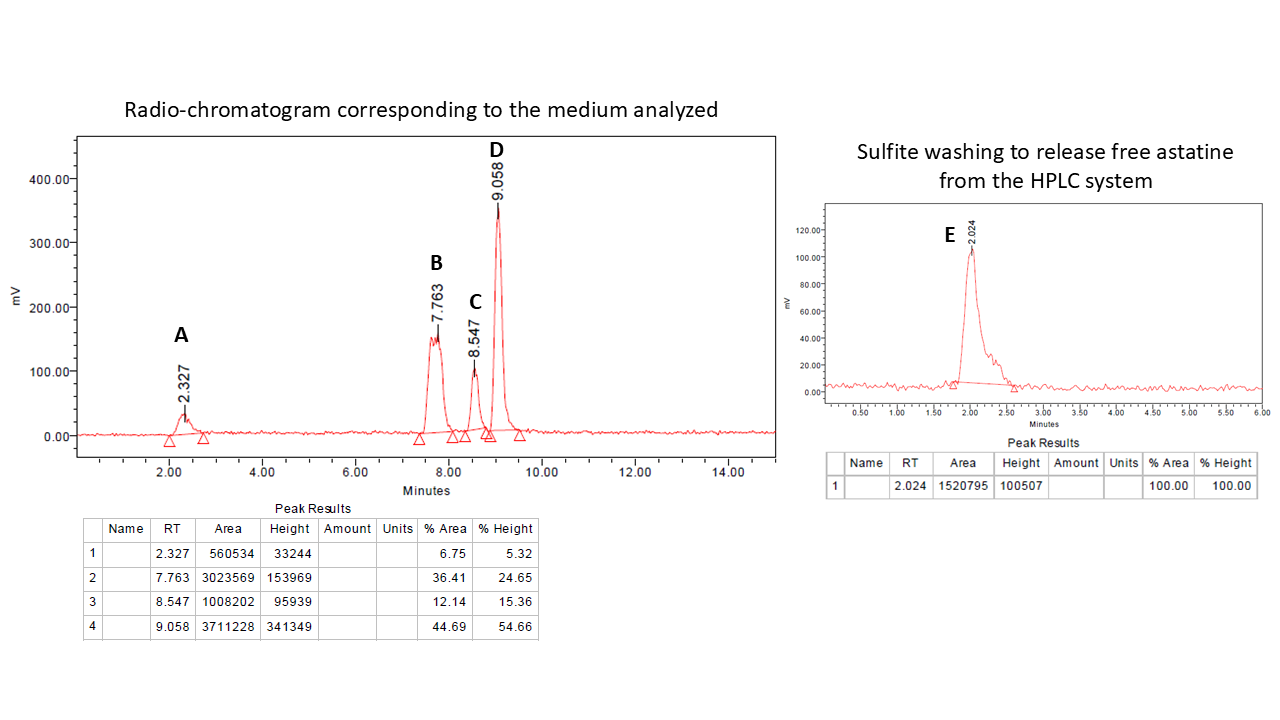


Figure S1. Example of [^211^At]AEB stability medium at 5 h in rat liver microsome

Each peak is then integrated, providing each peak area (noted A(A), A(B) etc…) to quantify the species present. Compound degradation (released astatine) during a stability assay was quantified by the ratio of free astatine (outgoing peak to injection peak (rt ≈ 2 min) when present (Figure S1 - A)) + sulfite washing (Figure S1 – E) to the sum of radio-chromatogram areas (free astatine area + area of peaks corresponding to astatoaryl compounds (Figure S1 – B, C and D)).

$$Released astatine \left( \% \right)=\frac{A \left( A \right)+A \left( E \right)}{A \left( A \right)+A \left( B \right)+A \left( C \right)+A \left( D \right)+A \left( E \right)}\times100= \frac{560534+1520795}{560534+3023569+1008202+3711228+1520795} \times100 \approx21 \%$$

Equation S1. Example of calculation of astatine released during stability

## II)Radio-chromatograms of radiolabeling medium

**3-[^211^At]astato-*N*-ethylbenzamide [^211^At]AEB (4)**

| Iodinated references | |
| --- | --- |
| 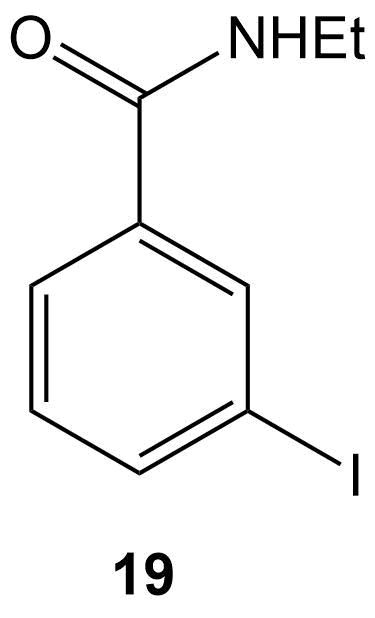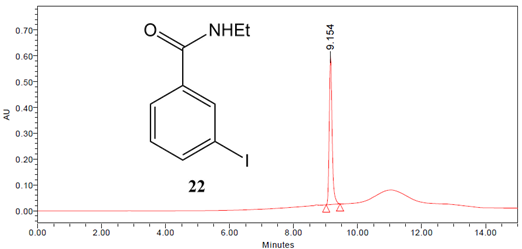 | **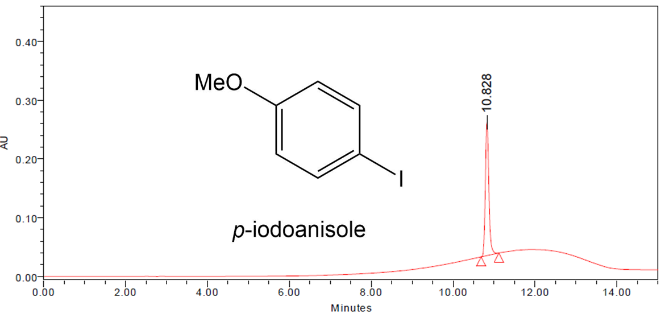** |
| Radiolabeling medium | **Sulfite washing** |
| 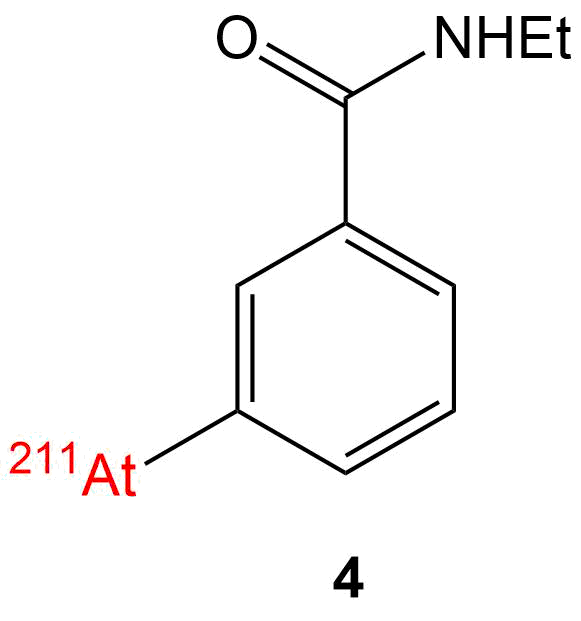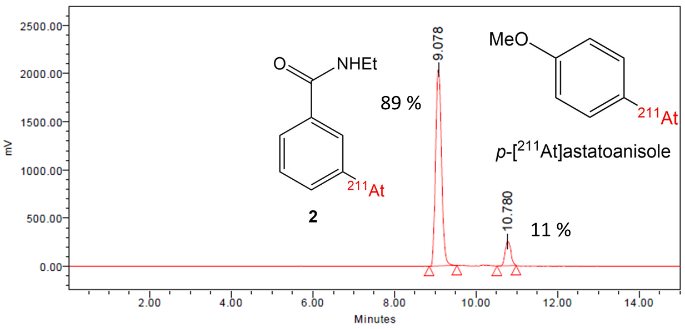 | **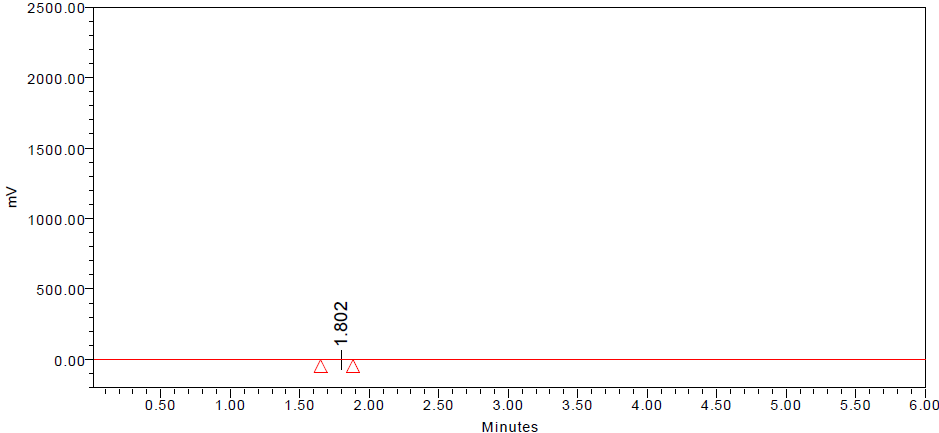**  Free At < 1% |
| Purification | |
| 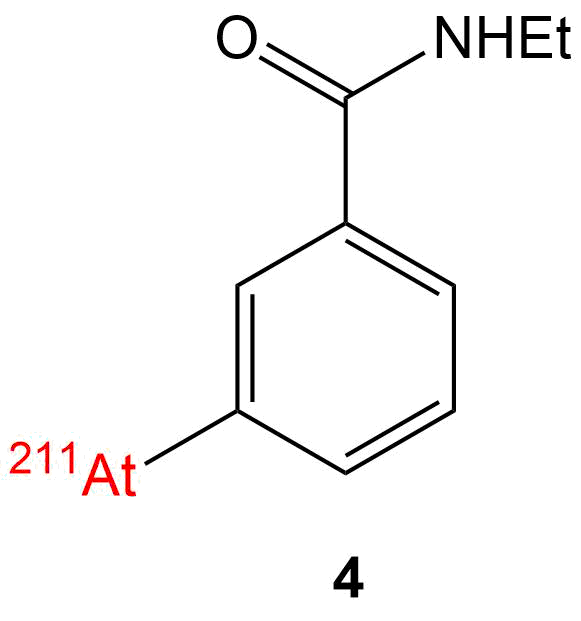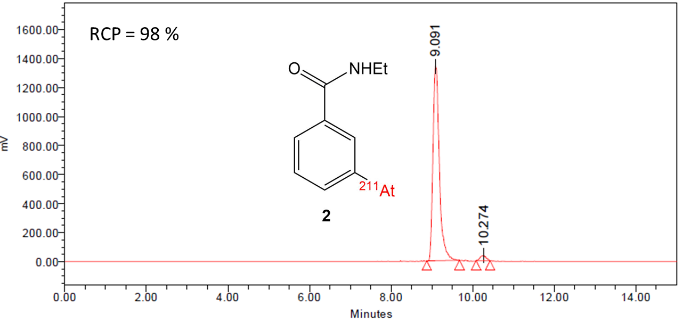 | **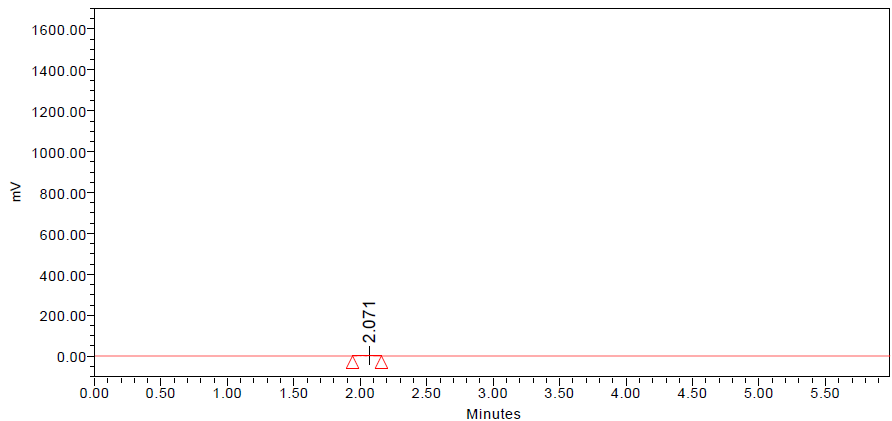**  Free At < 1% |

Figure S2. Iodinated references and radio-chromatograms of radiolabeling medium before and after purification of 4

**(2-[^211^At]astatophenyl)methanol (2)**

| Iodinated references | |
| --- | --- |
| 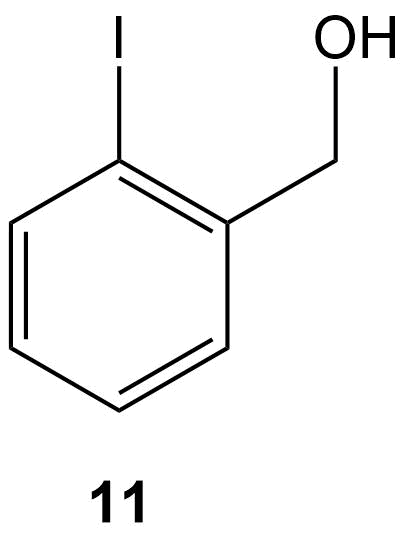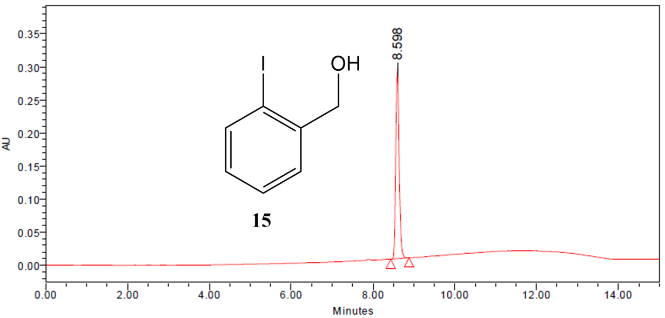 | **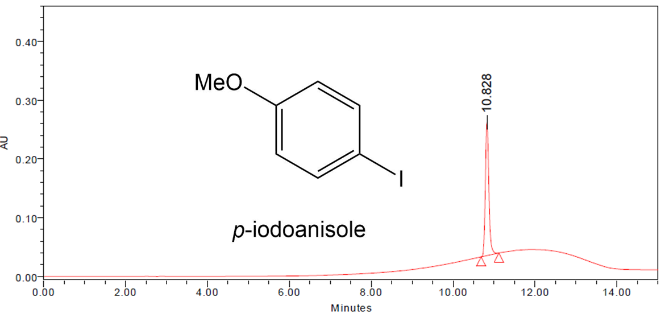** |
| Radiolabeling medium | **Sulfite washing** |
| 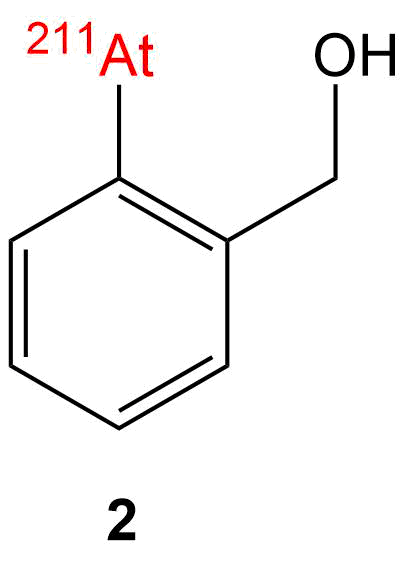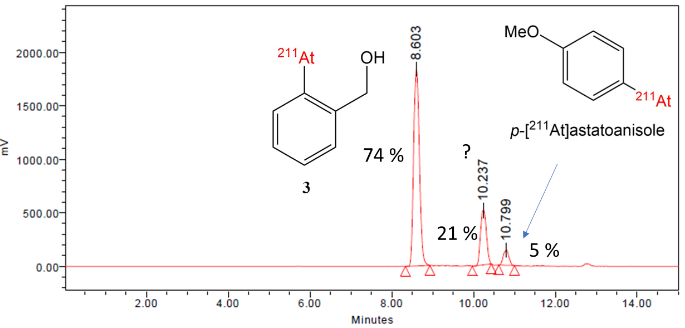 | **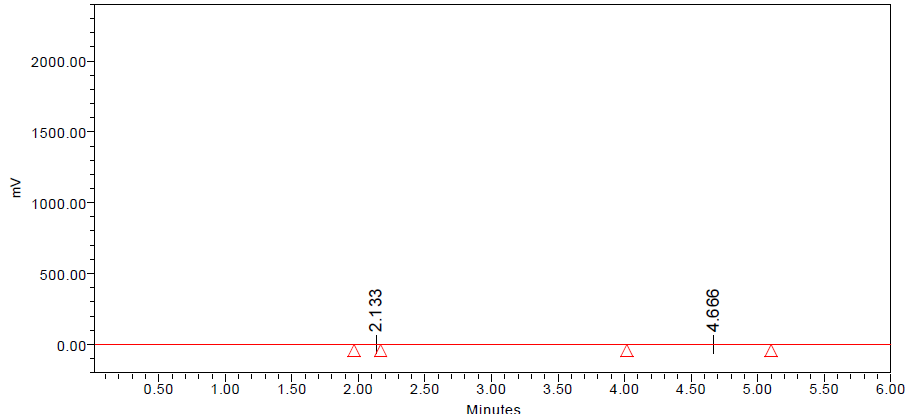**  Free At < 1% |
| Purification | |
| 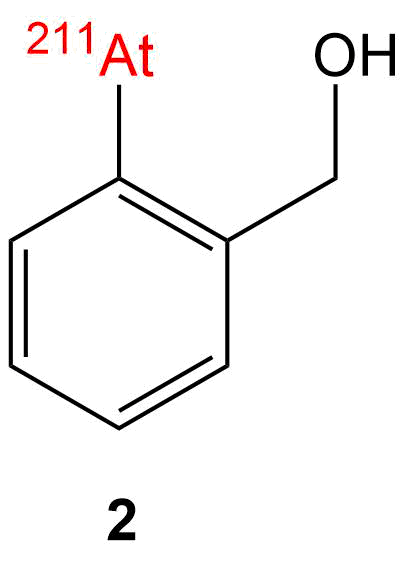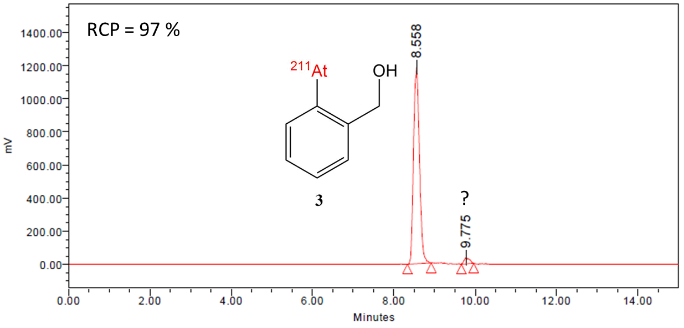 | **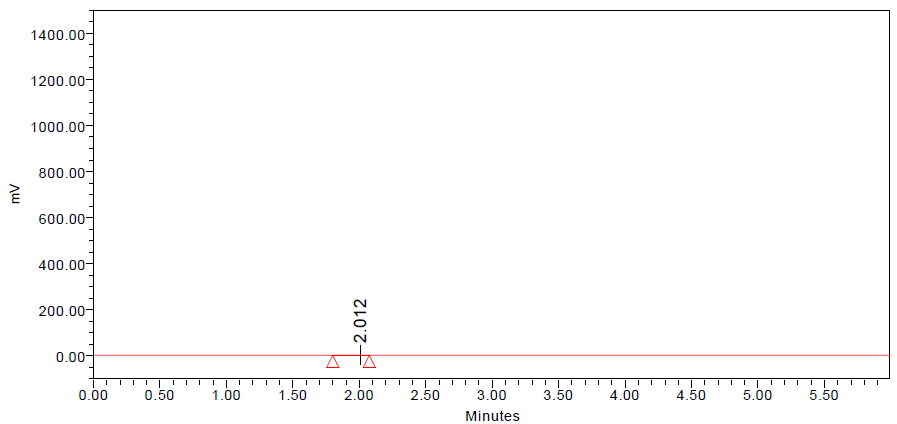**  Free At < 1% |

Figure S3. Iodinated references and radio-chromatograms of radiolabeling medium before and after purification of 2

**(2-[^211^At]astato-2,3-phenylene)dimethanol (3)**

| Iodinated references | |
| --- | --- |
| 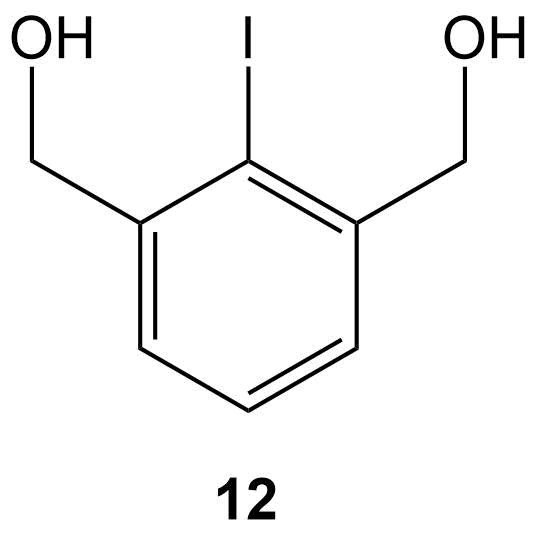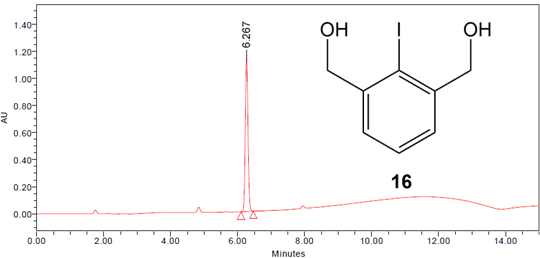 | **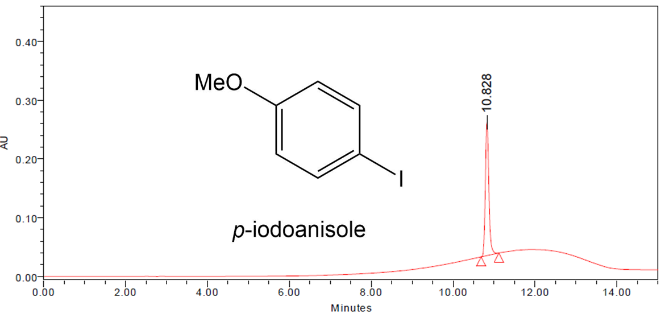** |
| Radiolabeling medium | **Sulfite washing** |
| 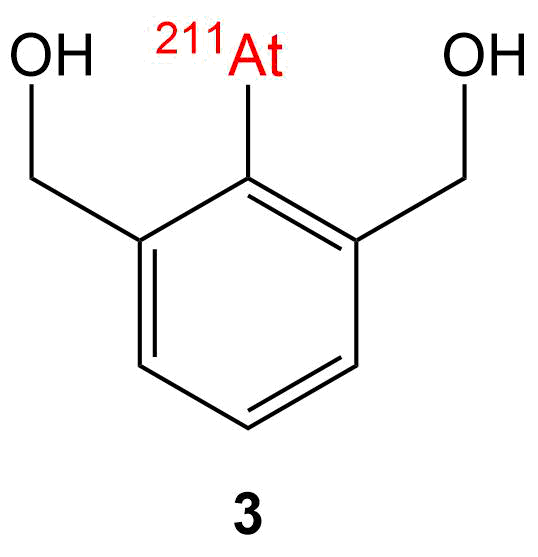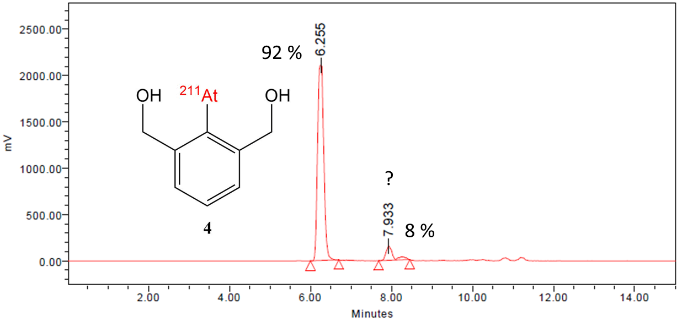 | **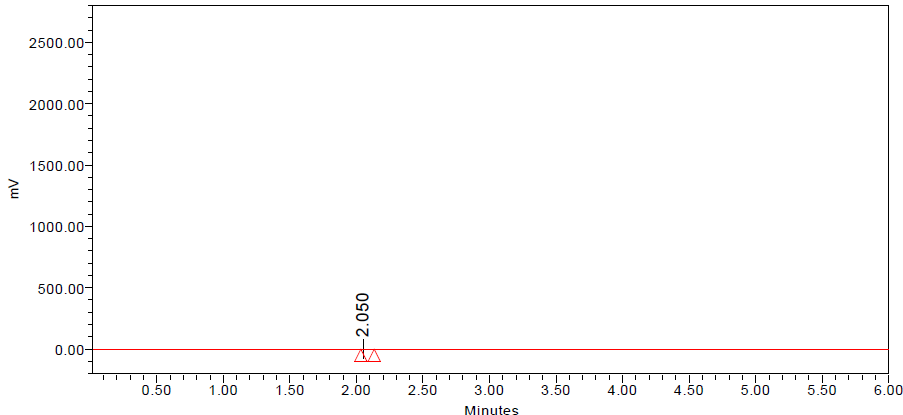**  Free At < 1% |
| Purification | |
| 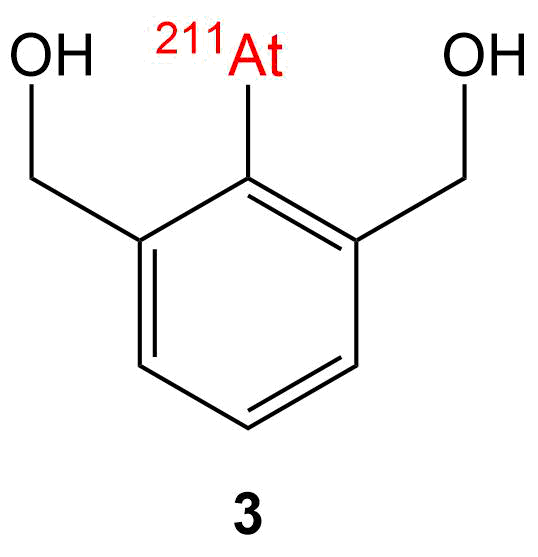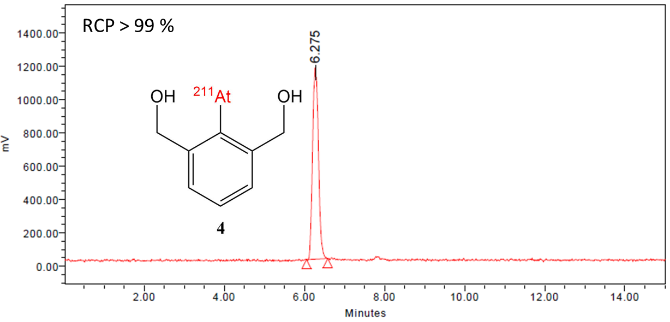 | **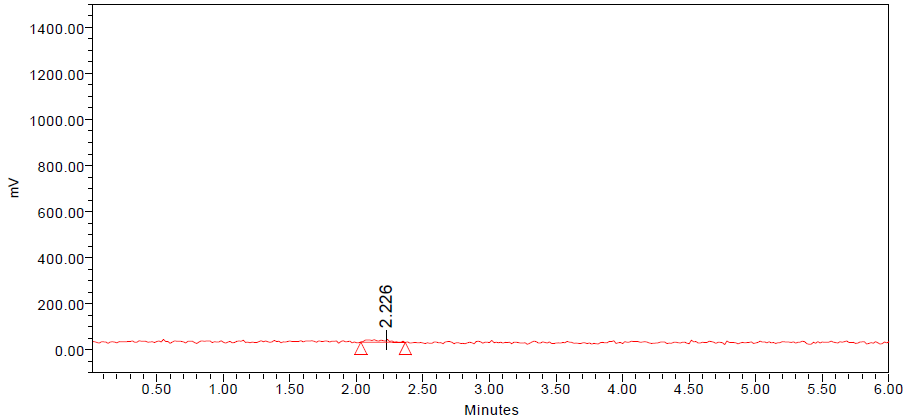**  Free At < 1% |

Figure S4. Iodinated references and radio-chromatograms of radiolabeling medium before and after purification of 3

## III) Radio-chromatograms of stability medium

| Stability medium analysis | Sulfite washing |
| --- | --- |
| 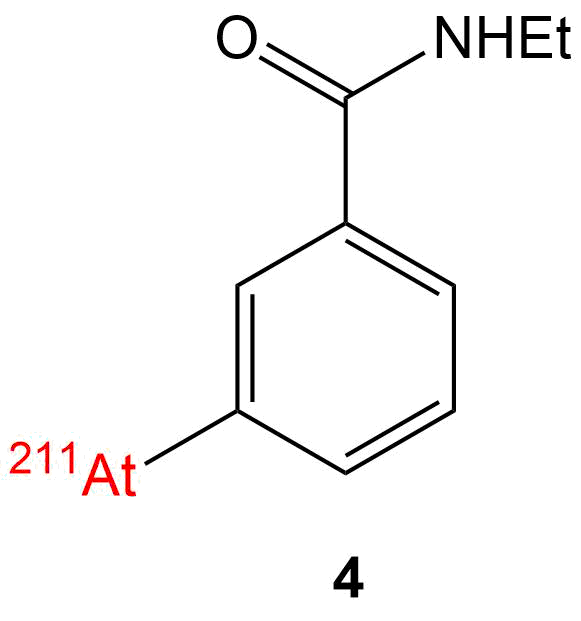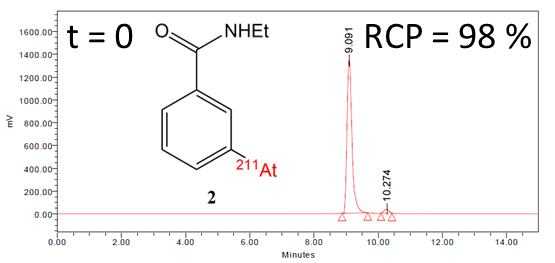 | **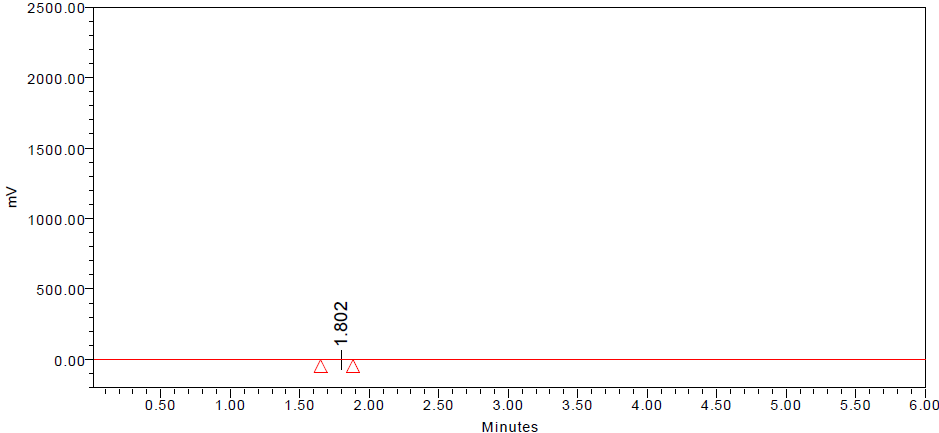**  Free At < 1% |
| 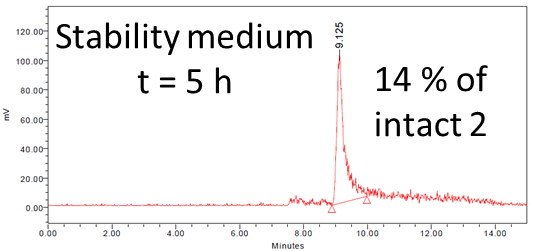 | 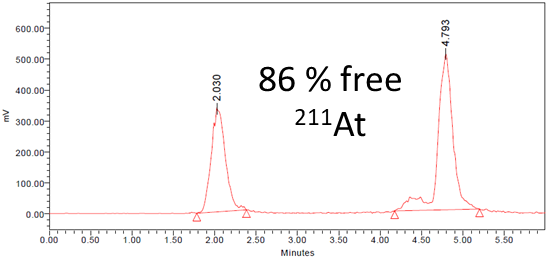 |
| 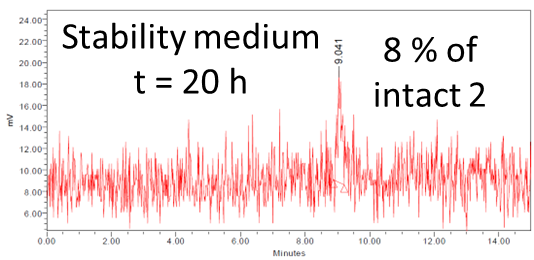 | 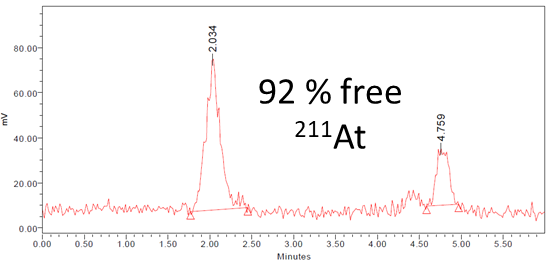 |

Figure S5. HPLC monitoring of [^211^At]AEB stability in oxidizing/acidic media (KMnO_4_ 1 mM – acetate buffer pH 4.7 50 mM)

| Stability medium analysis | Sulfite washing |
| --- | --- |
| 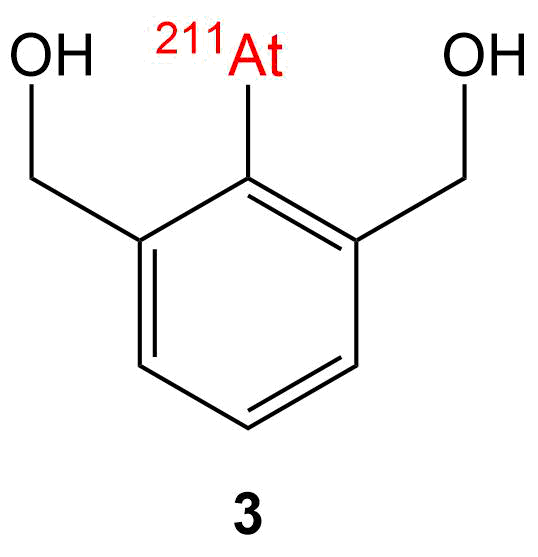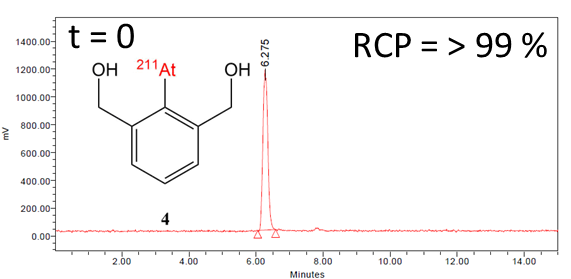 | **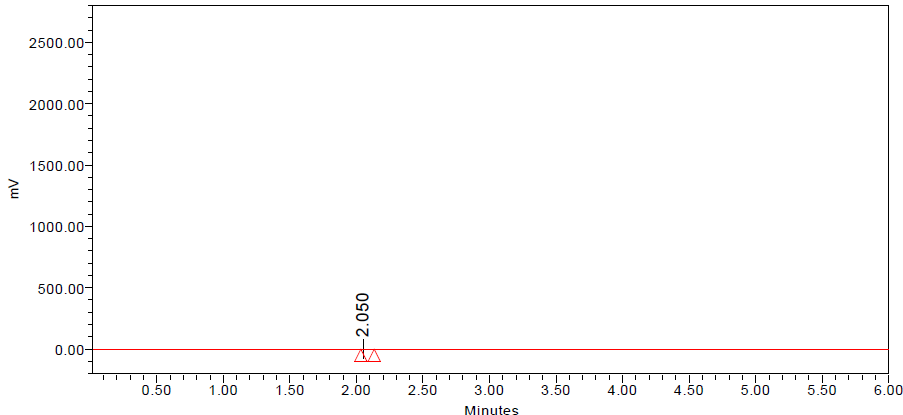**  Free At < 1% |
| 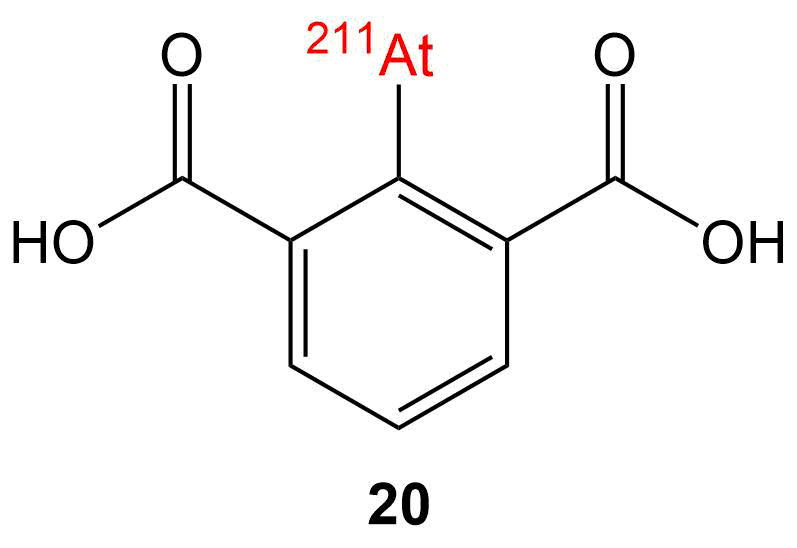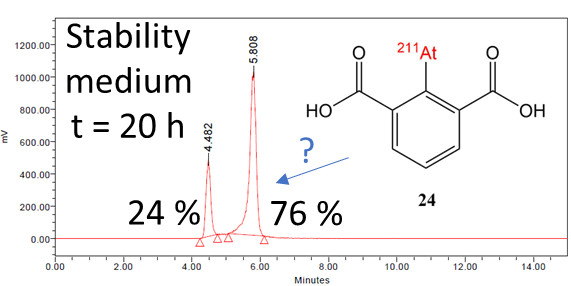 | 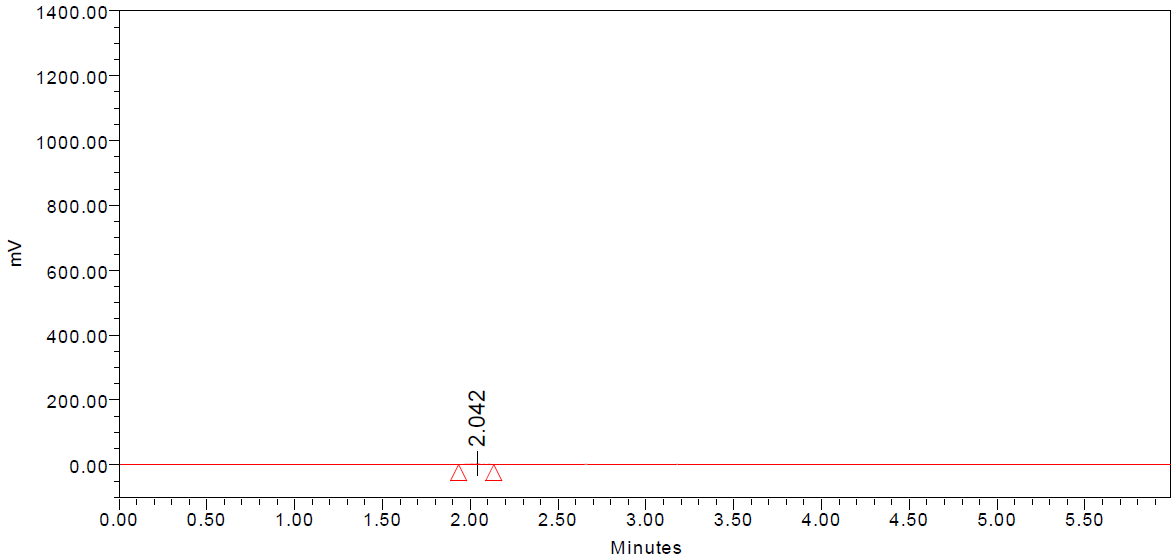  Free At < 1% |
| 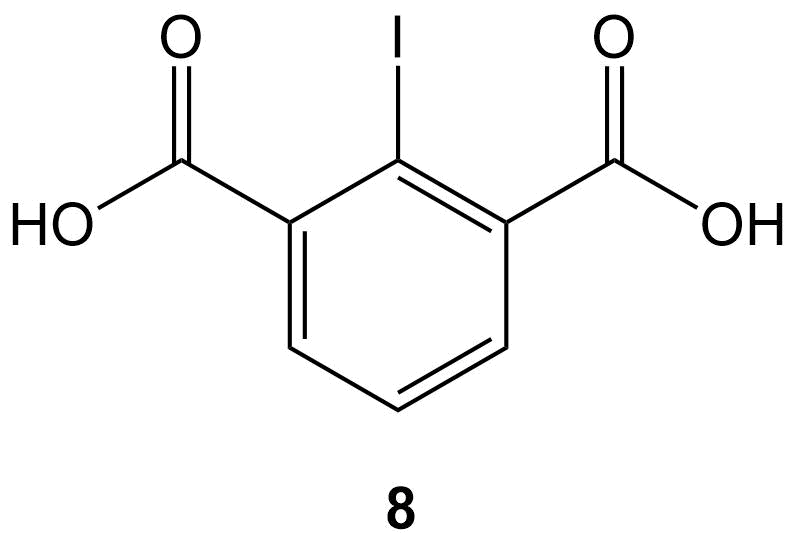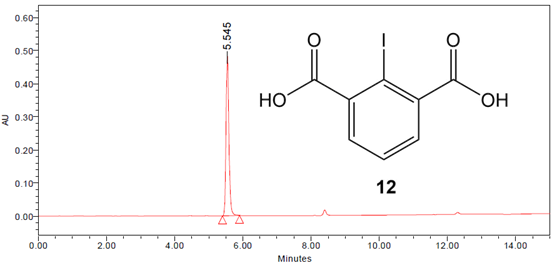 | - |

Figure S6. HPLC monitoring of 3 stability in oxidizing/acidic media (KMnO_4_ 1 mM – acetate buffer pH 4.7 50 mM)

Free At < 1%

| Stability medium analysis | Sulfite washing |
| --- | --- |
| 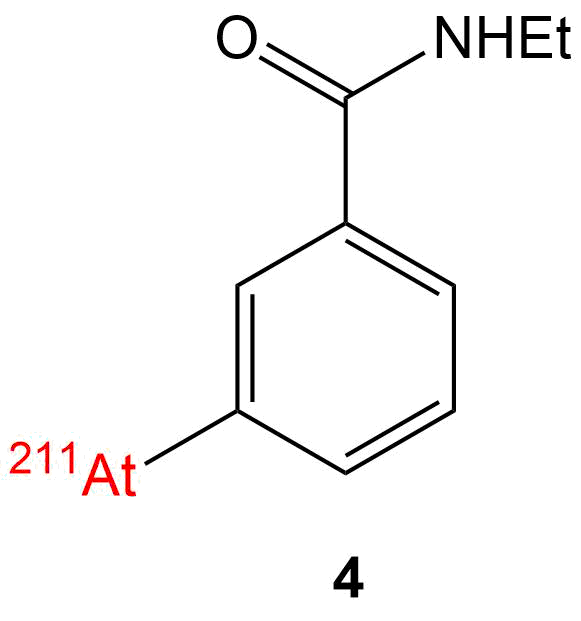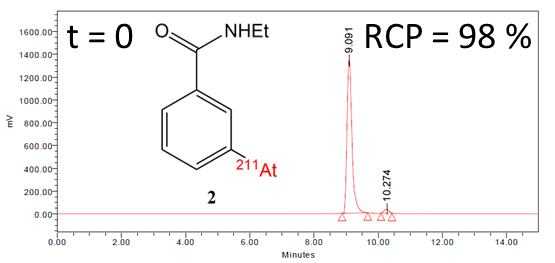 | **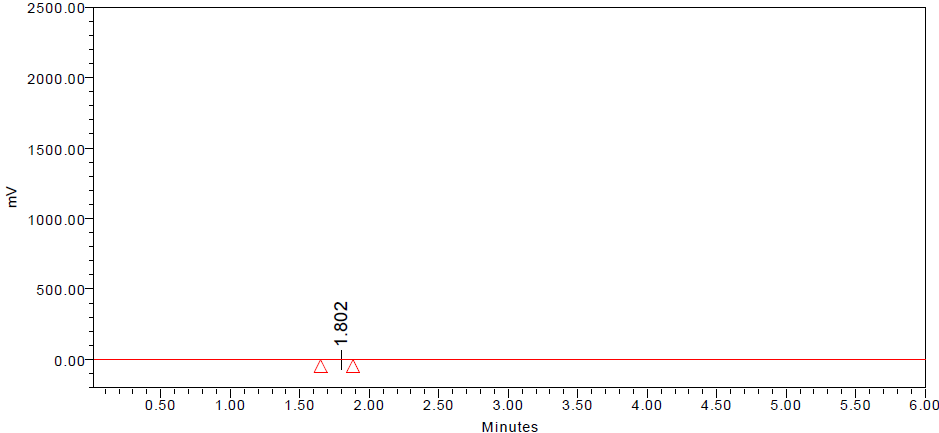** |
| 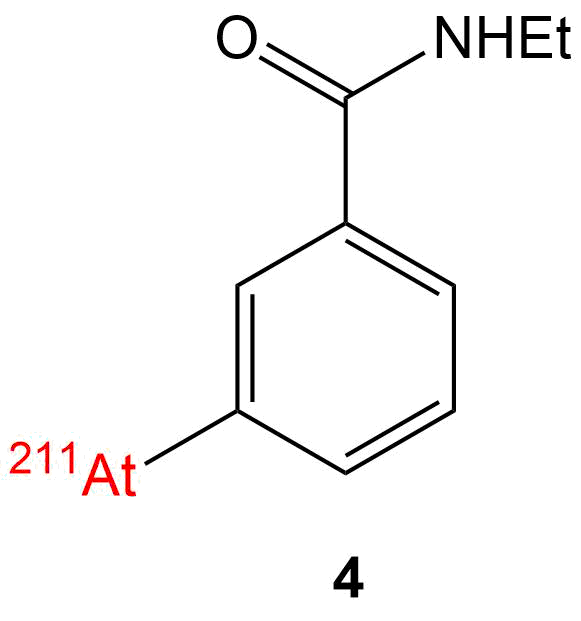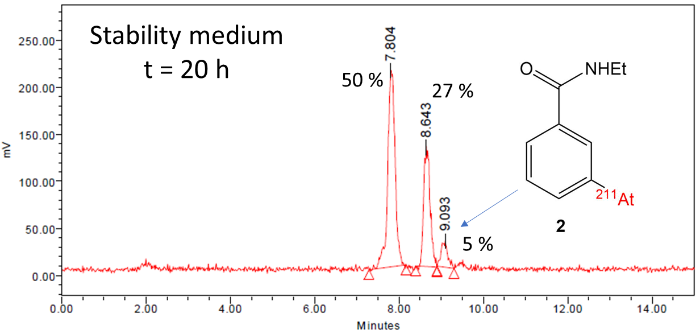 | 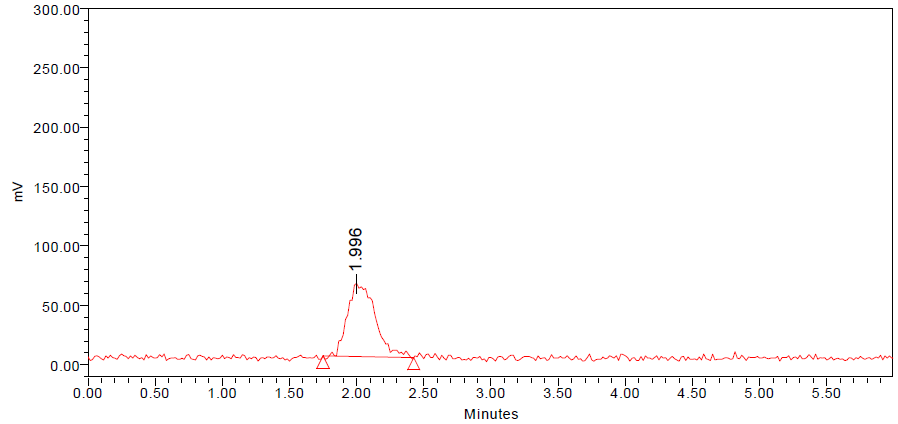  Free At 18% |

Figure S7. HPLC monitoring of 4 stability in rat liver microsome

| Stability medium analysis | Sulfite washing |
| --- | --- |
| 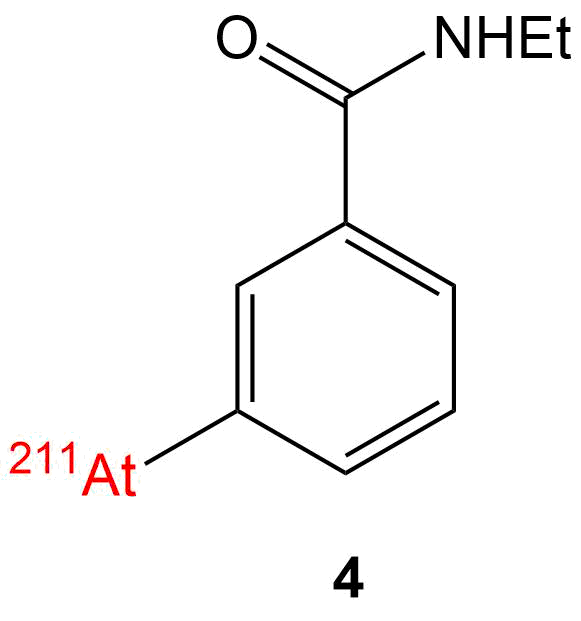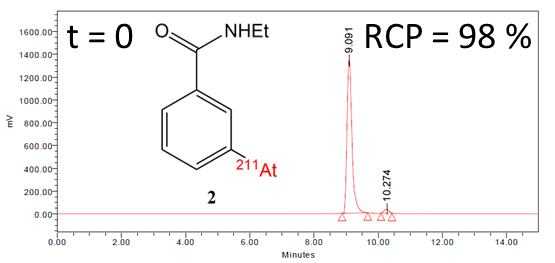 | **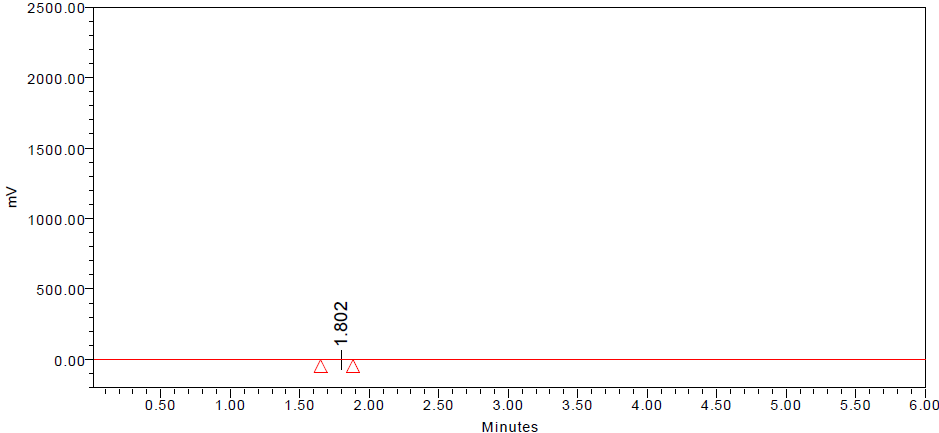**  Free At < 1% |
| 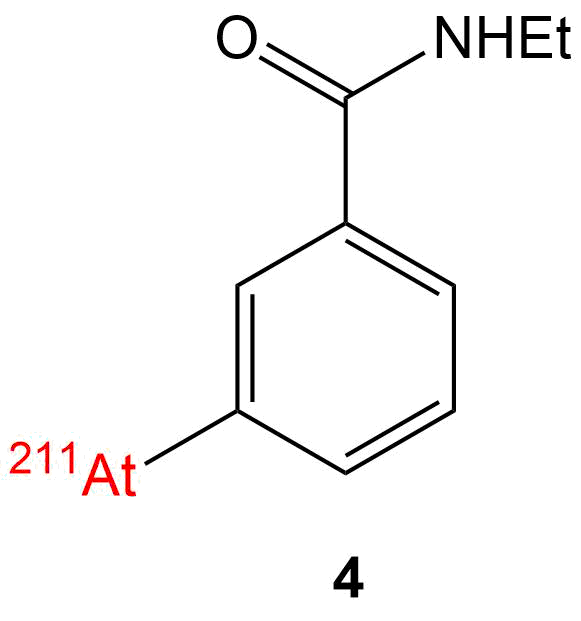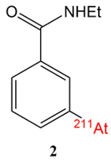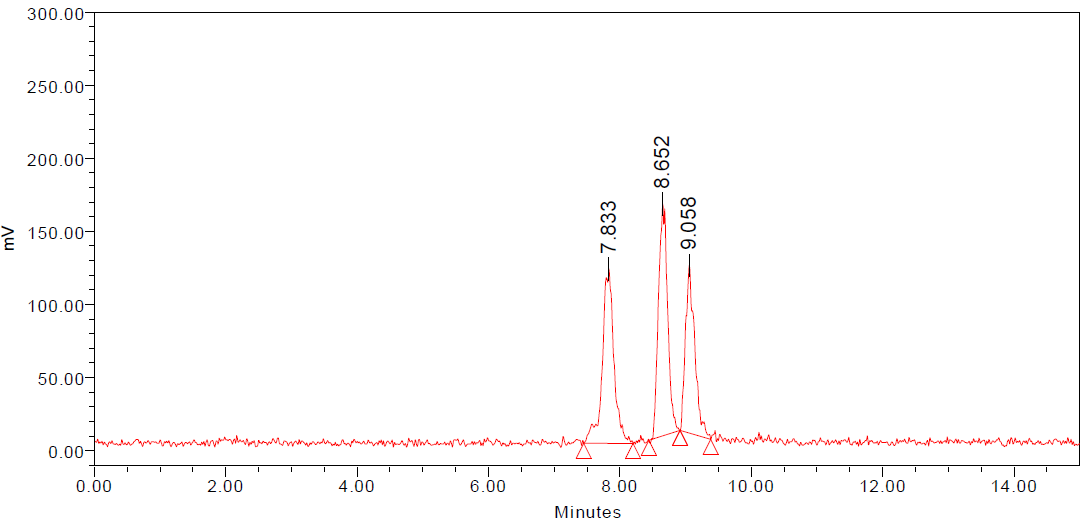  Stability medium t = 20 h  25%  34%  32% | 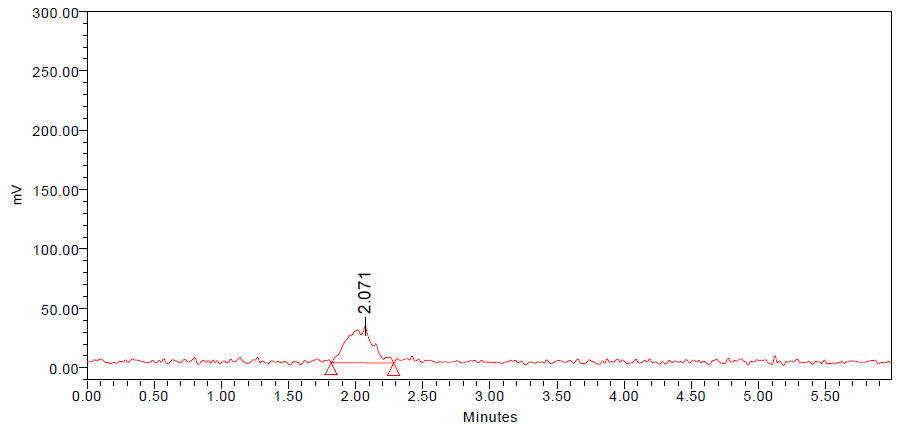  Free At 9% |

Figure S8. HPLC monitoring of 4 stability in human liver microsome

| Stability medium analysis | Sulfite washing |
| --- | --- |
| 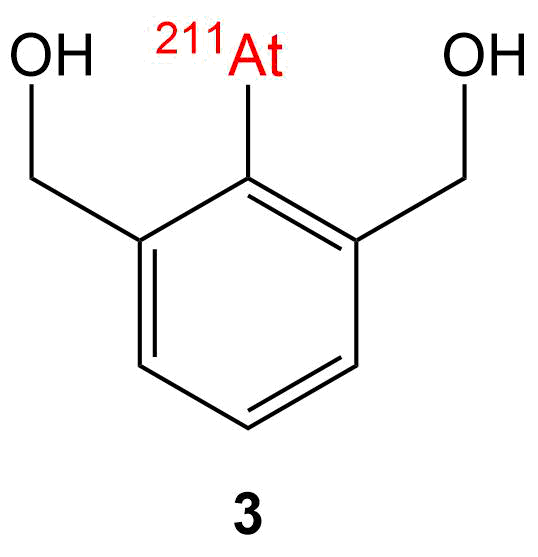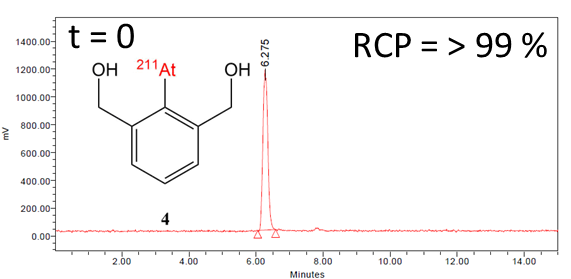 | **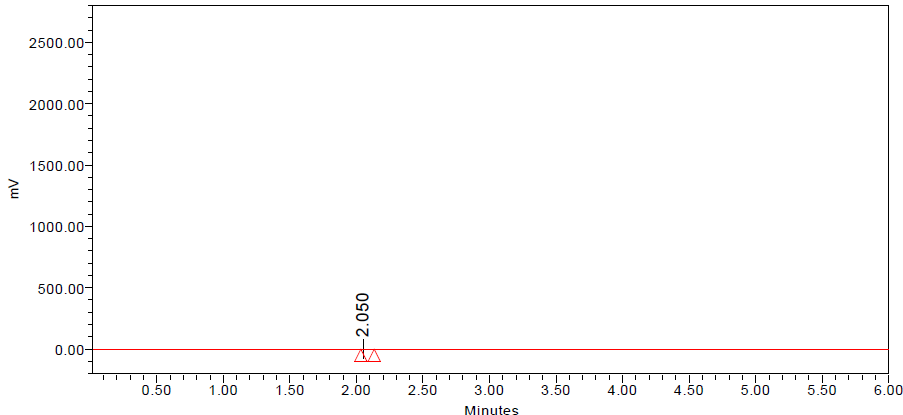** |
| 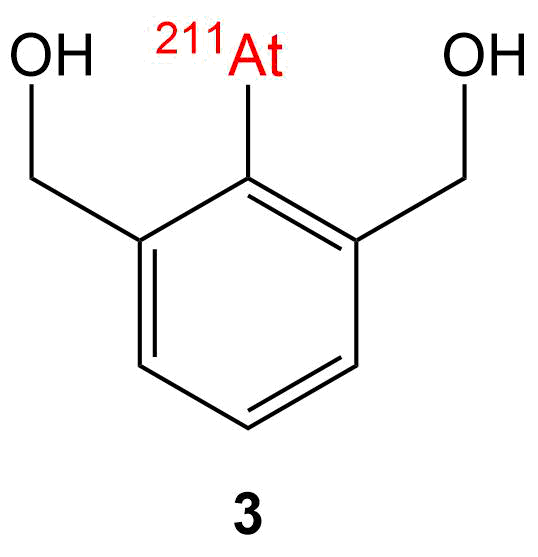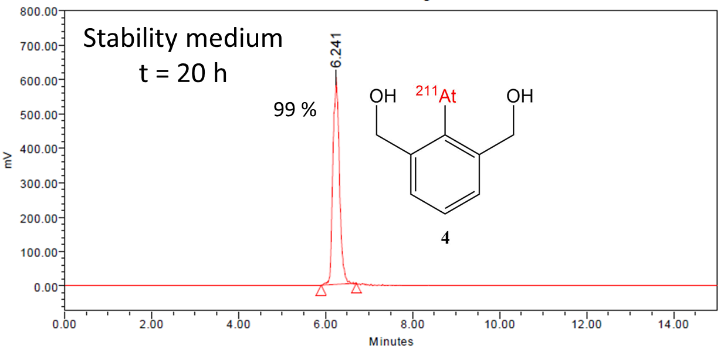 | 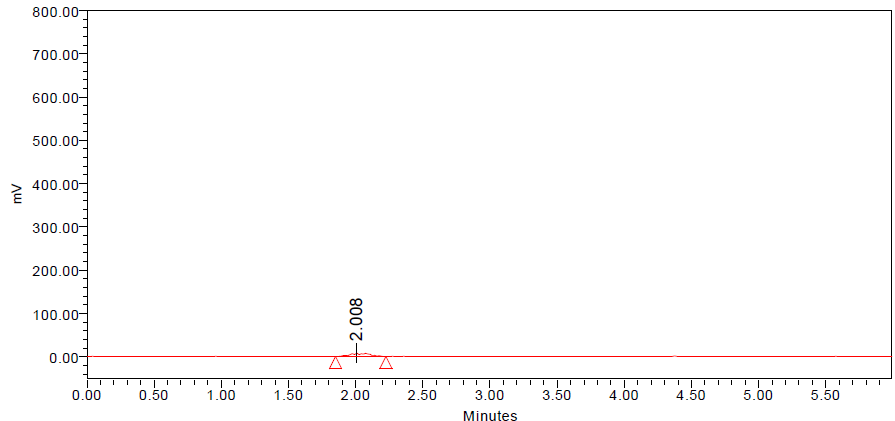  Free At 1% |

Free At < 1%

Figure S9. HPLC monitoring of 3 stability in rat liver microsome

| Stability medium analysis | Sulfite washing |
| --- | --- |
| 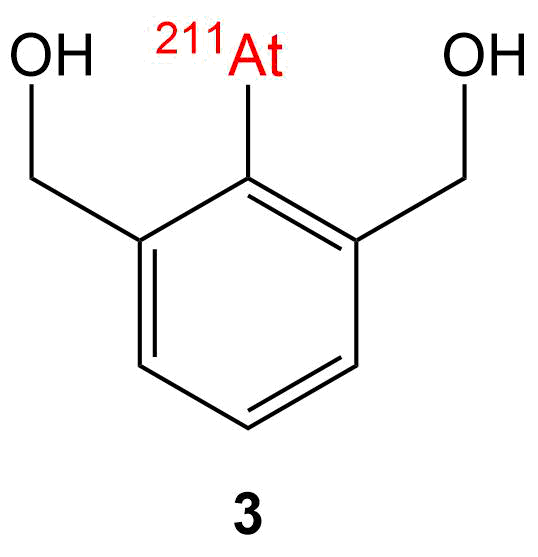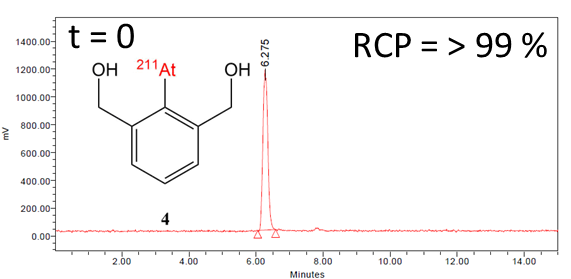 | **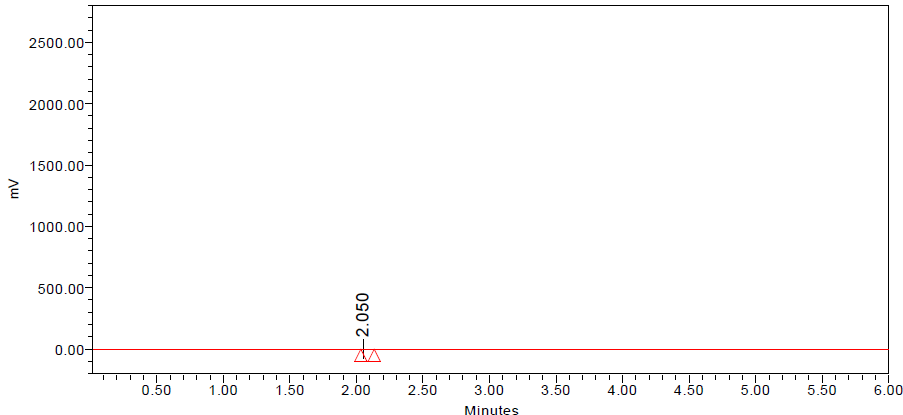**  Free At < 1% |
| 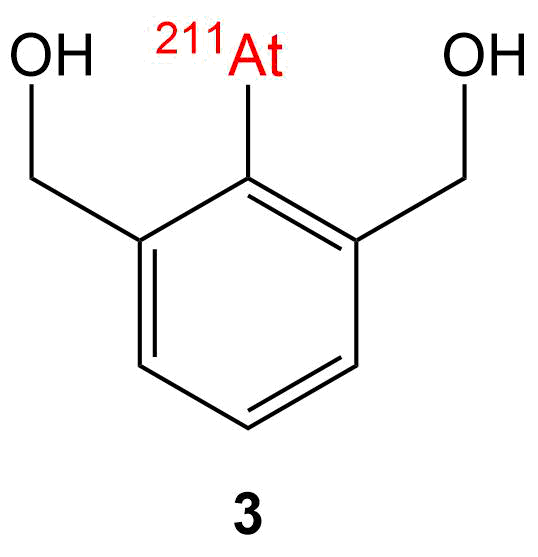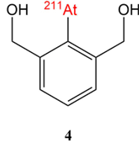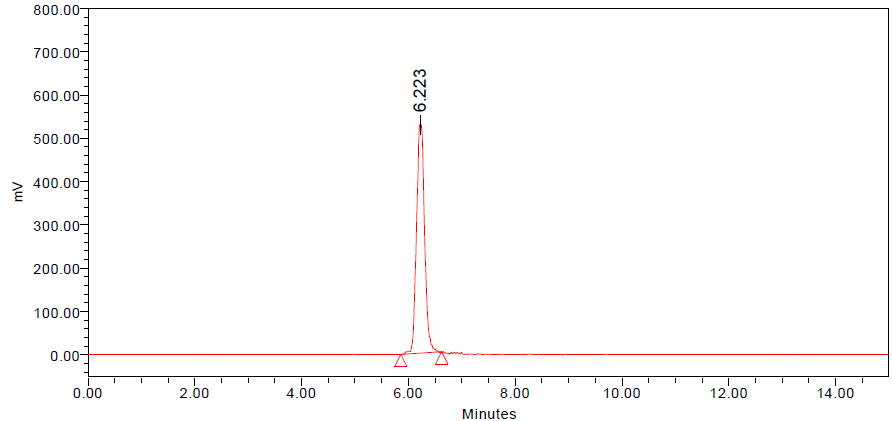  Stability medium t = 20 h  99% | 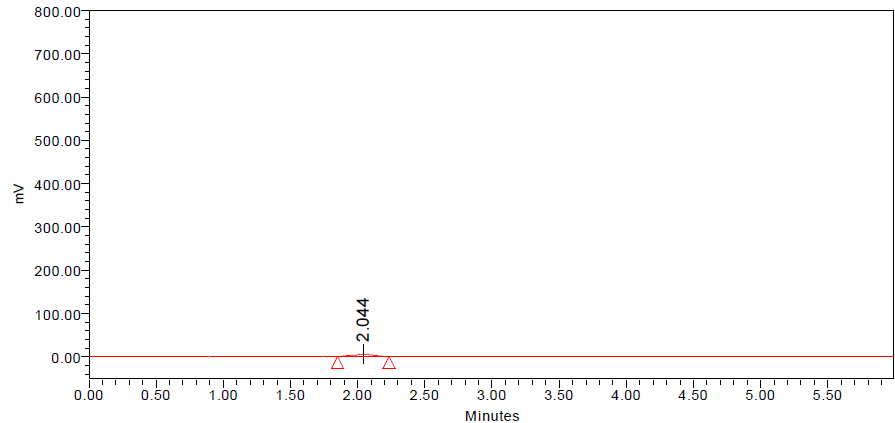  Free At 1% |

Figure S10. HPLC monitoring of 3 stability in human liver microsome

## IV) Stability of [^211^At]astatobenzene in rat liver microsome

Figure S11. Monitoring of deastatination of [^211^At]astatobenzene in rat liver microsome at 30 min, 1, 2, 4, 7 and 24 h (n = 1)

## V) Analysis of synthesized molecules

**2-iodo-benzoic acid (7)**


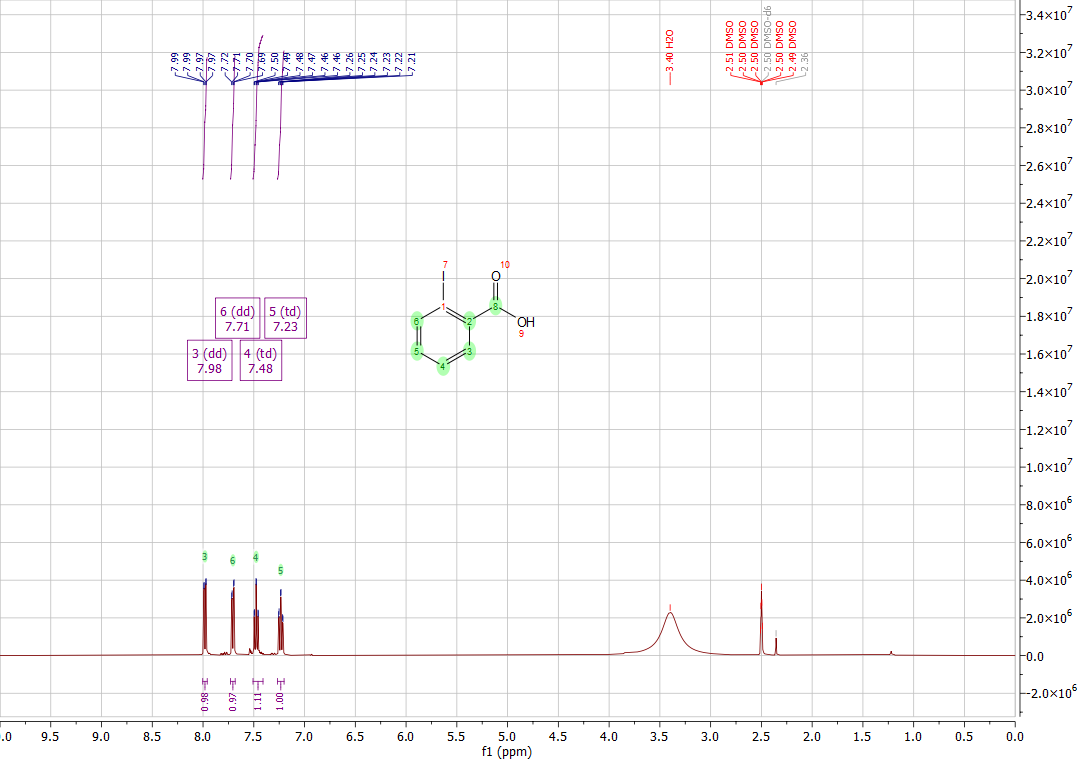


Figure S12. ^1^H NMR spectrum of compound 7


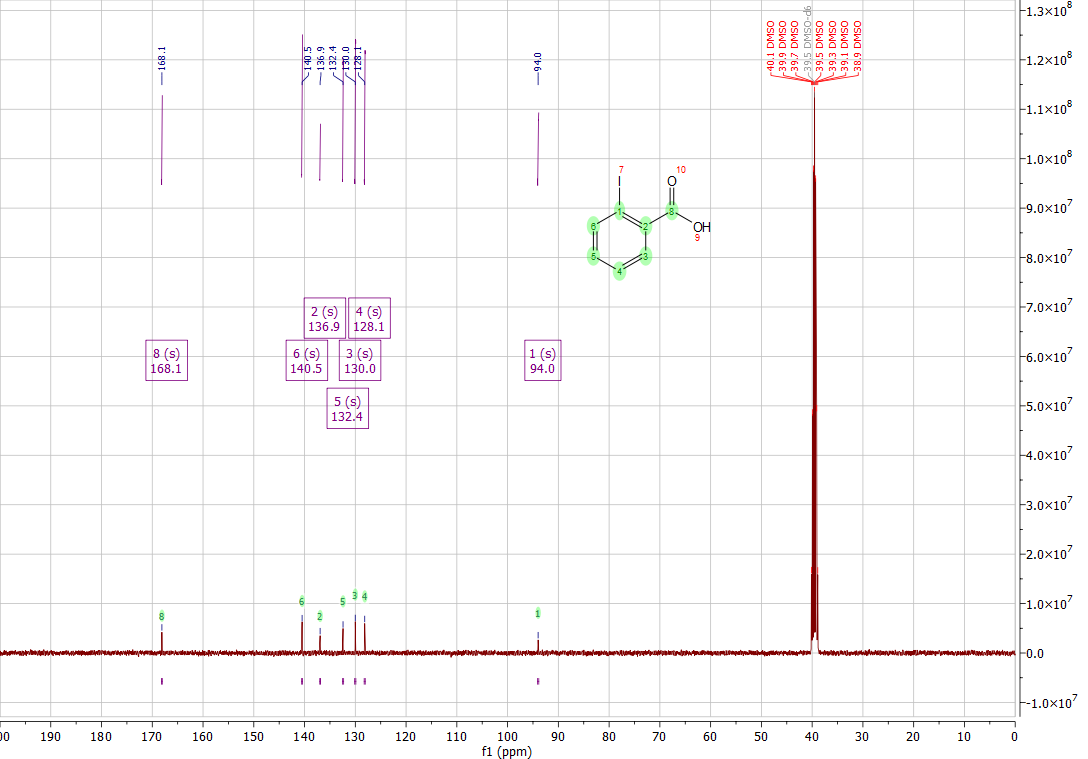


Figure S13. ^13^C NMR spectrum of compound 7

**2-iodo-isophtalic acid (8)**

**
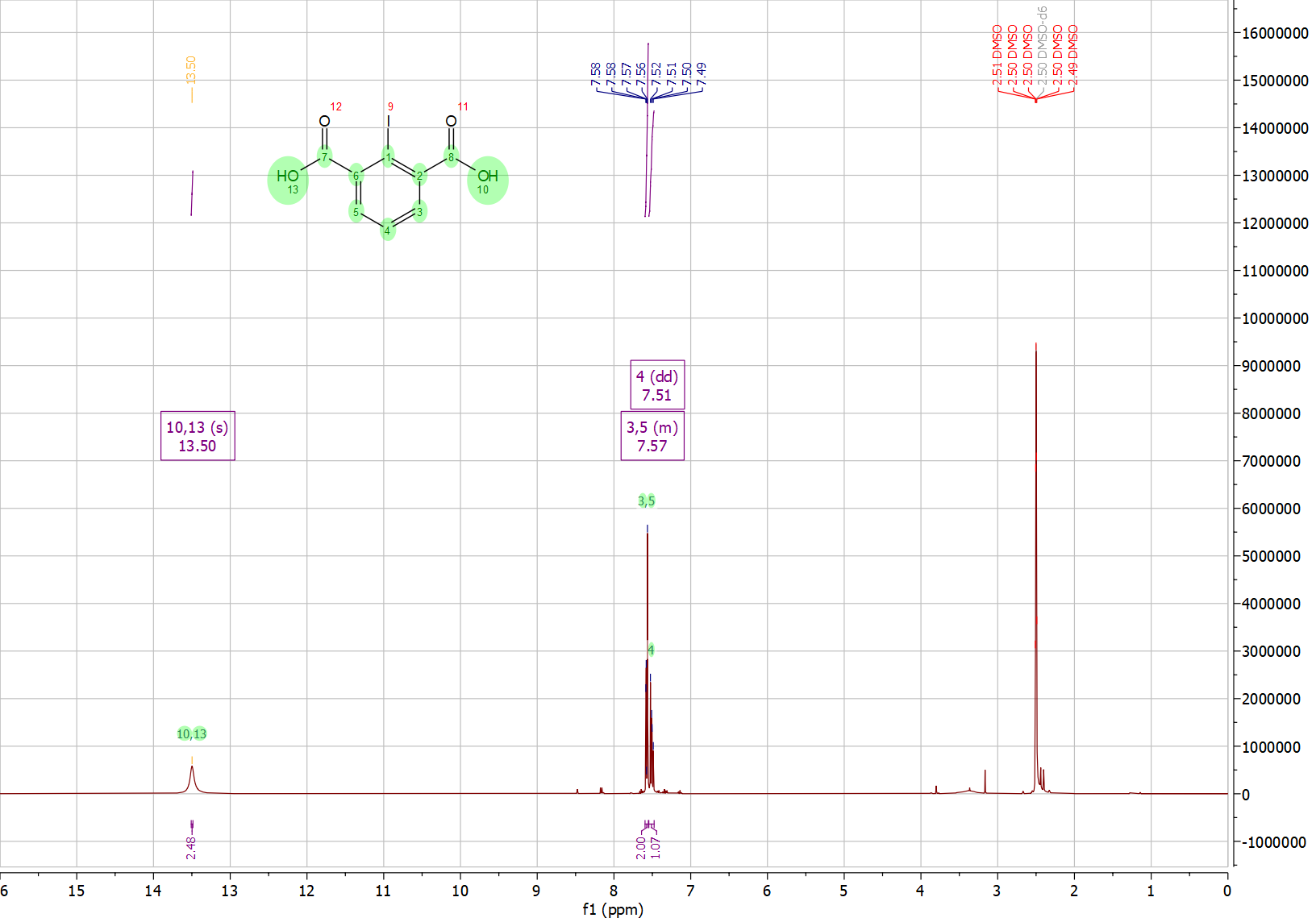
**

Figure S14. ^1^H NMR spectrum of compound 8

**
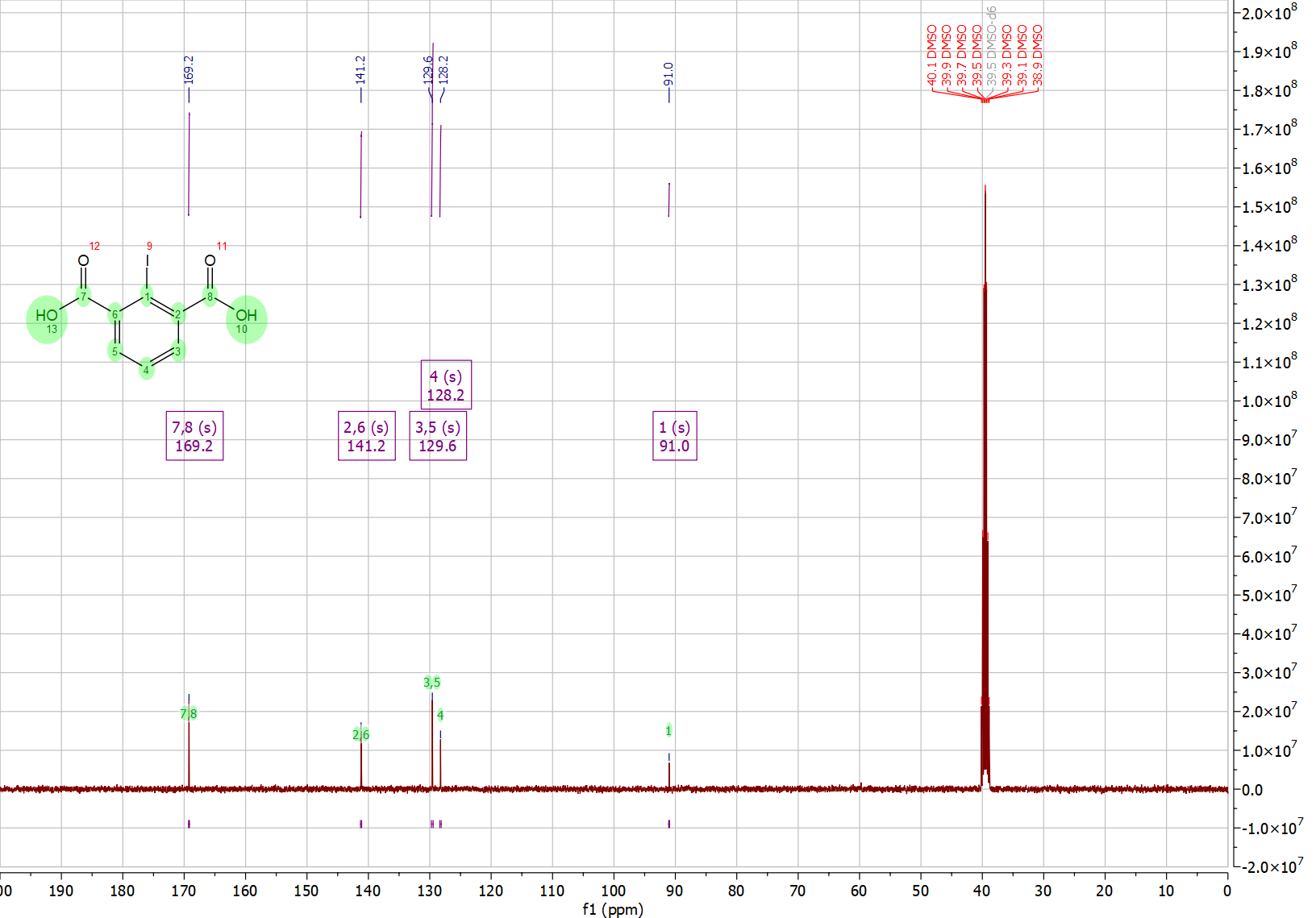
**

Figure S15. ^13^C NMR spectrum of compound 8

**2-iodo-benzoic acid methyl ester (9)**


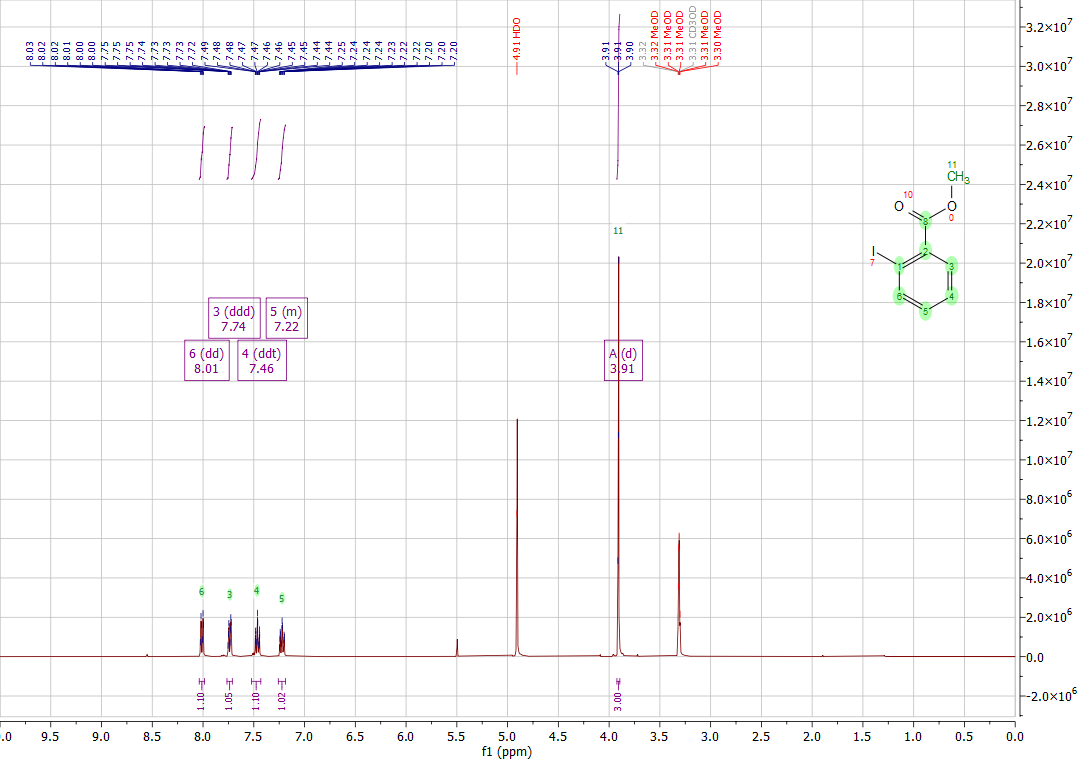


Figure S16. ^1^H NMR spectrum of compound 9


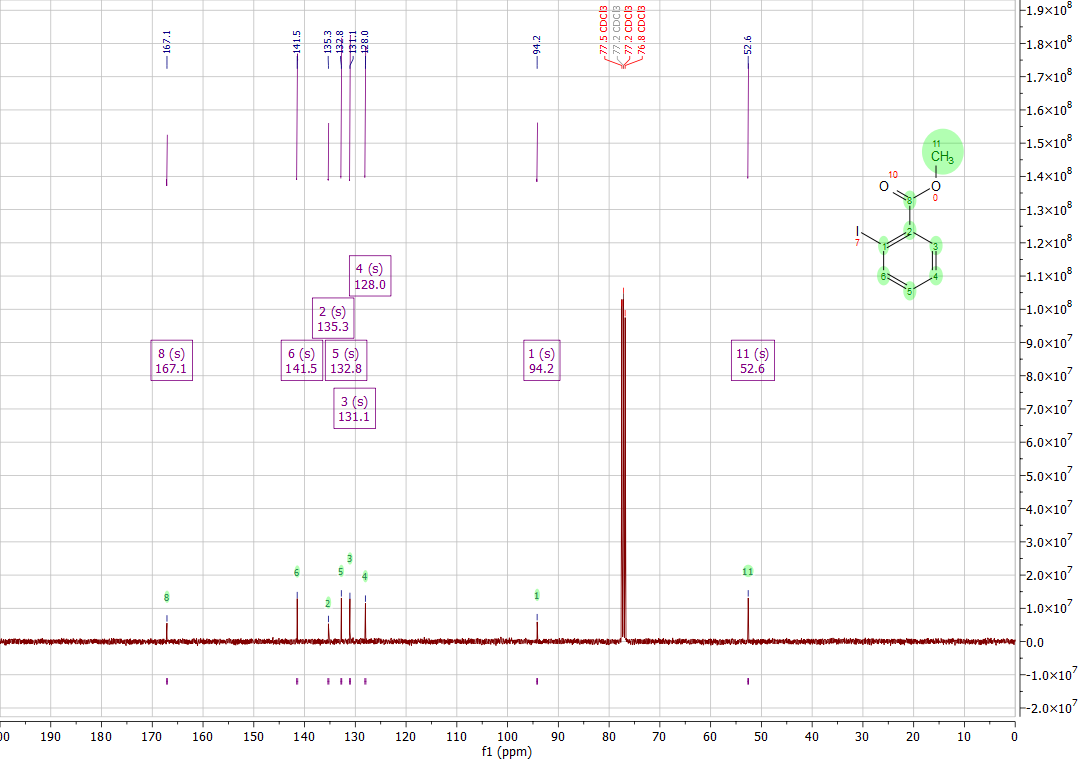


Figure S17. ^13^C NMR spectrum of compound 9

**2-iodo-isophtalic acid dimethyl ester (10)**


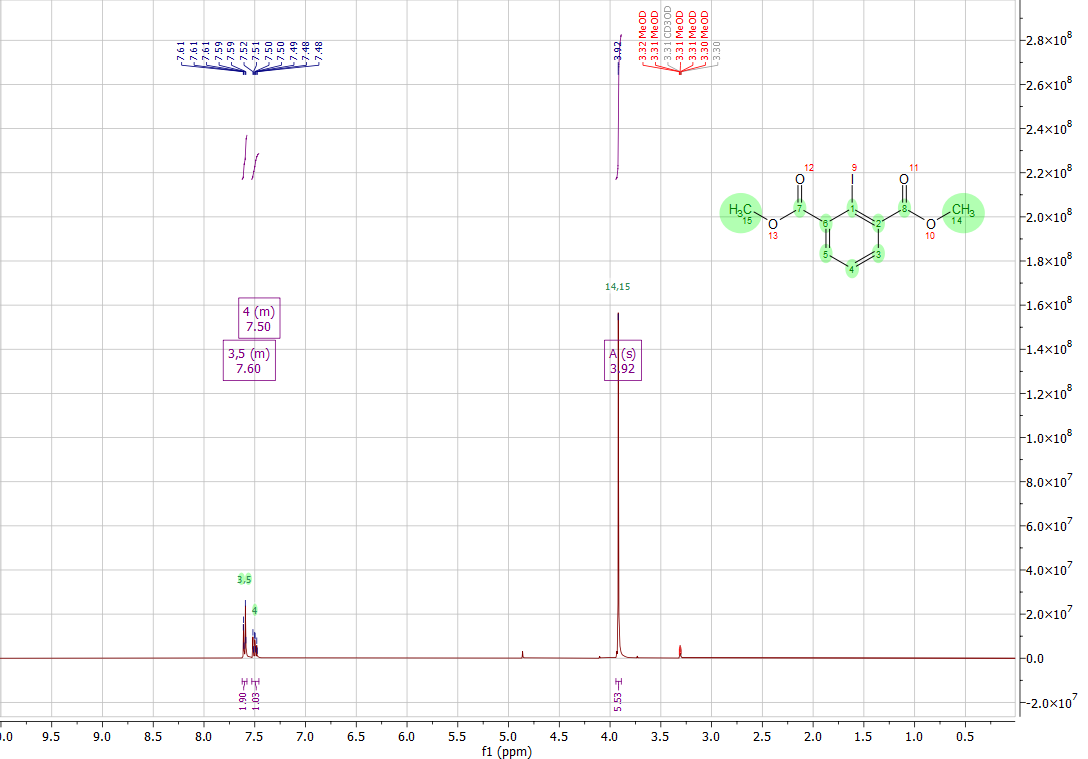


Figure S18. ^1^H NMR spectrum of compound 10


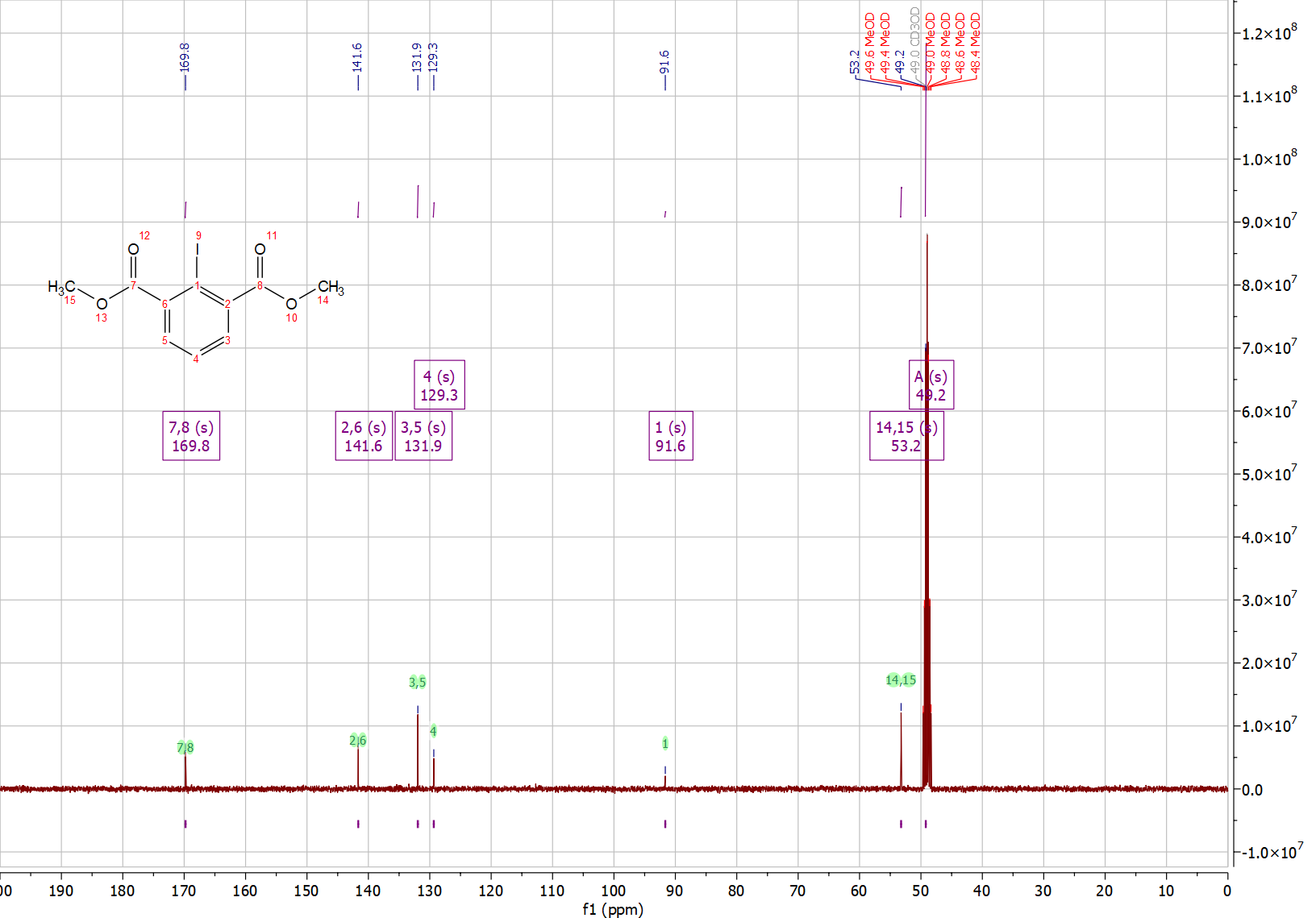


Figure S19. ^13^C NMR spectrum of compound 10

**(2-iodo-phenyl)-methanol (11)**


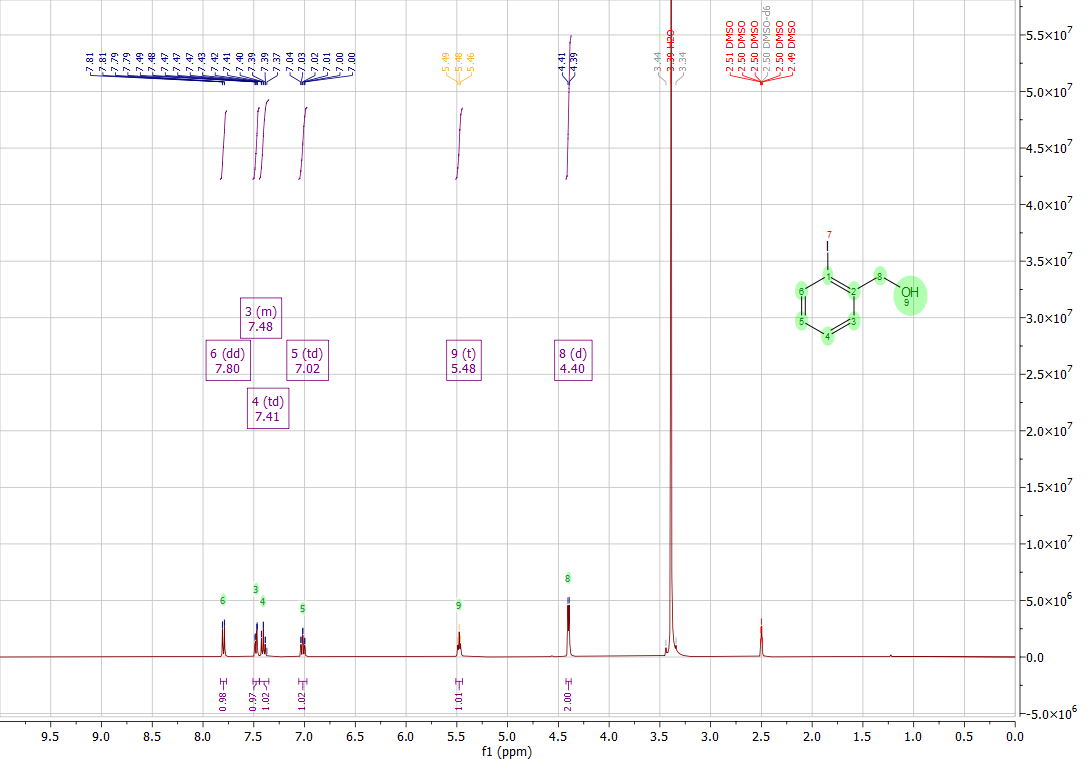


Figure S20. ^1^H NMR spectrum of compound 11


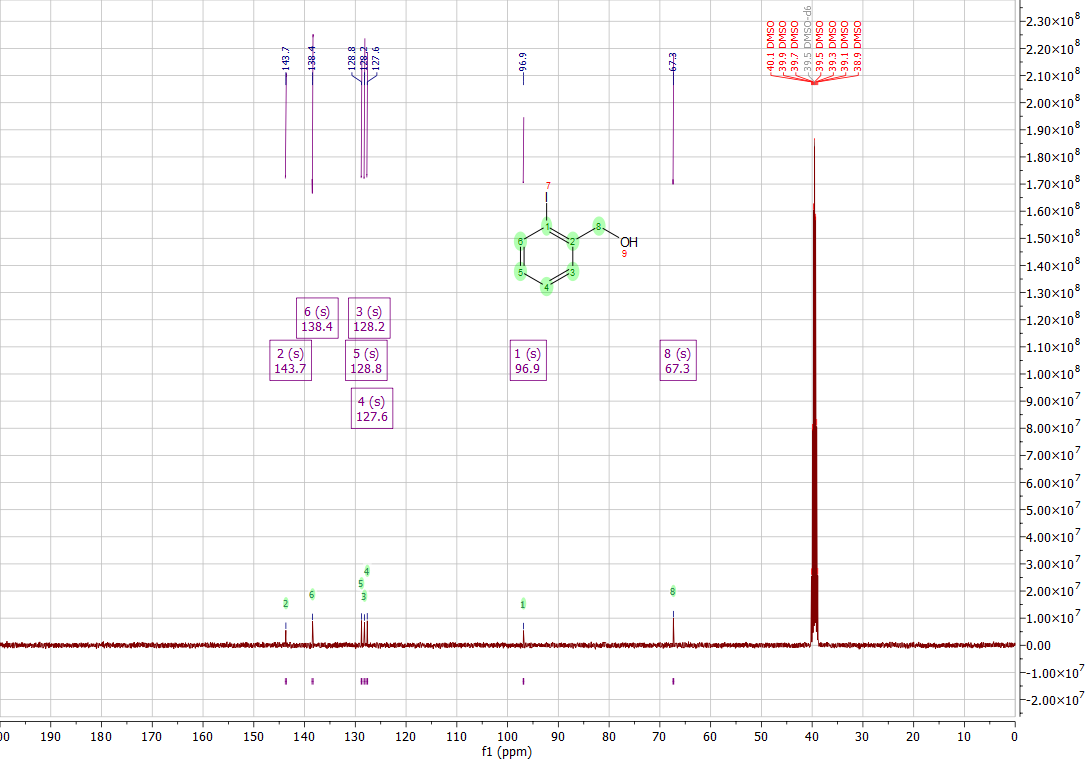


Figure S21. ^13^C NMR spectrum of compound 11

**(3-hydroxymethyl-2-iodo-phenyl)-methanol (12)**


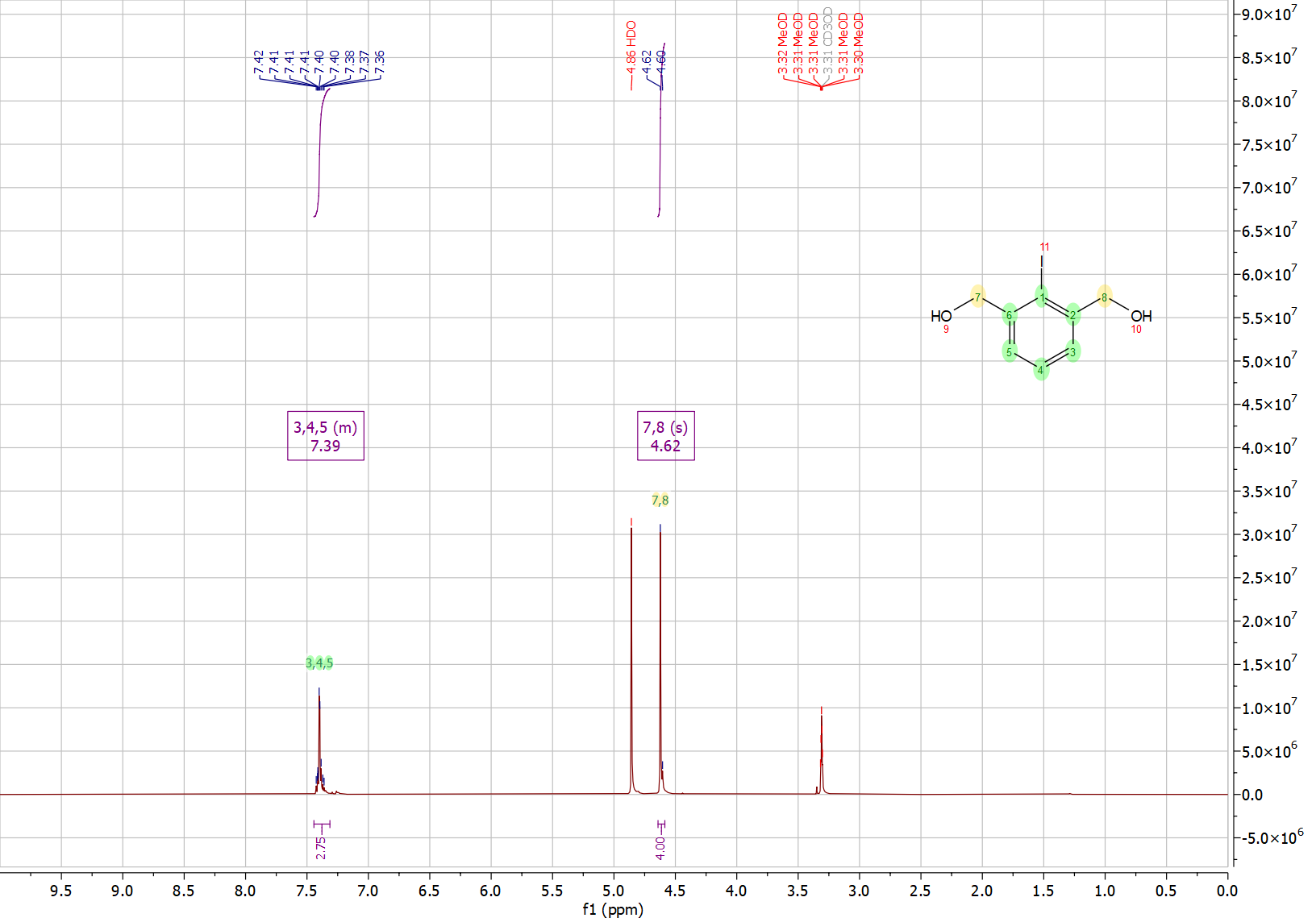


Figure S22. ^1^H NMR spectrum of compound 12


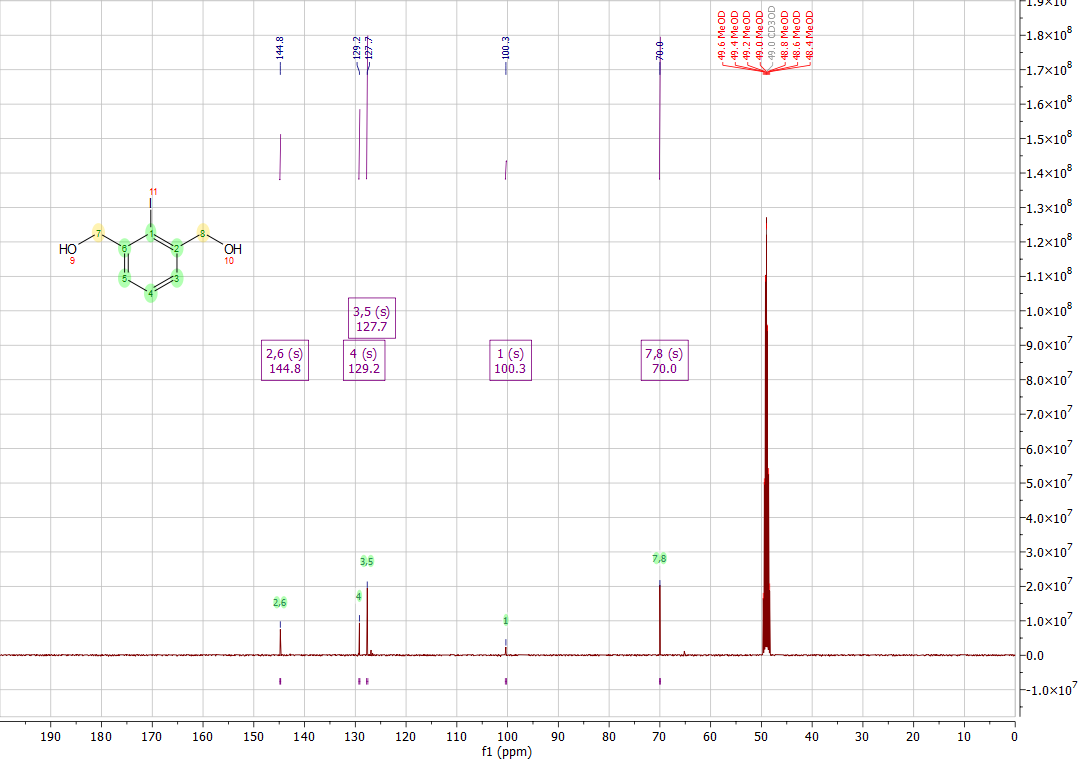


Figure S23. ^13^C NMR spectrum of compound 12

**Acetic acid 2-iodo-benzyl ester (13)**


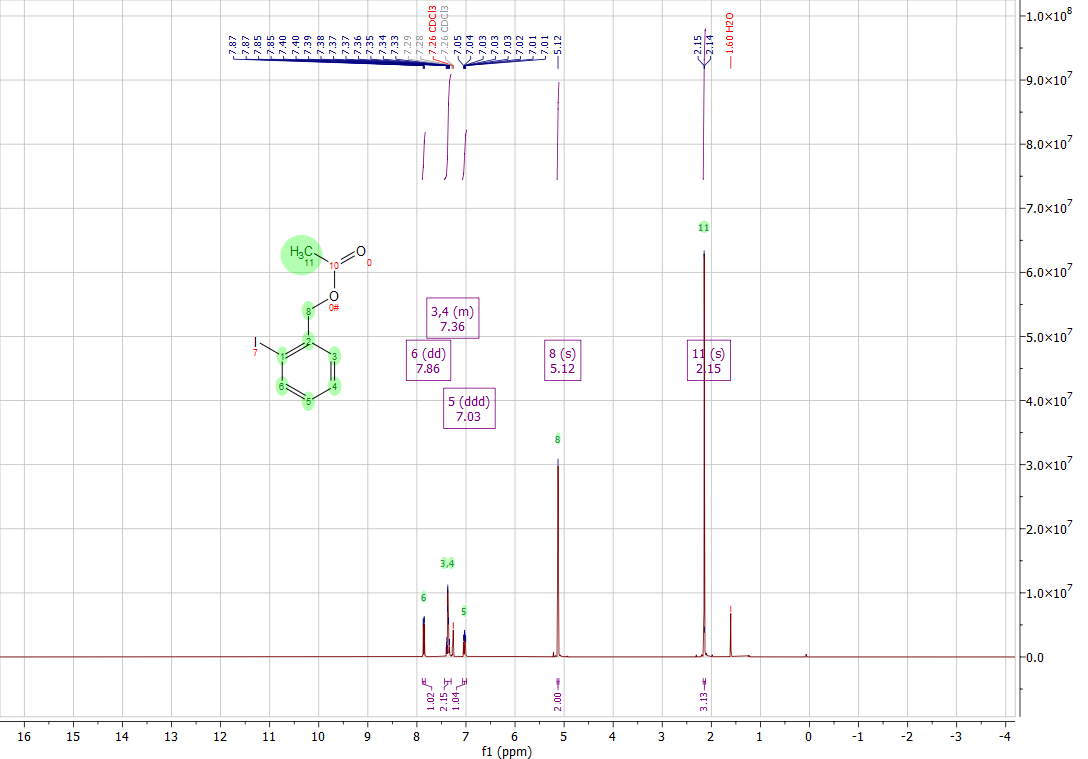


Figure S24. ^1^H NMR spectrum of compound 13


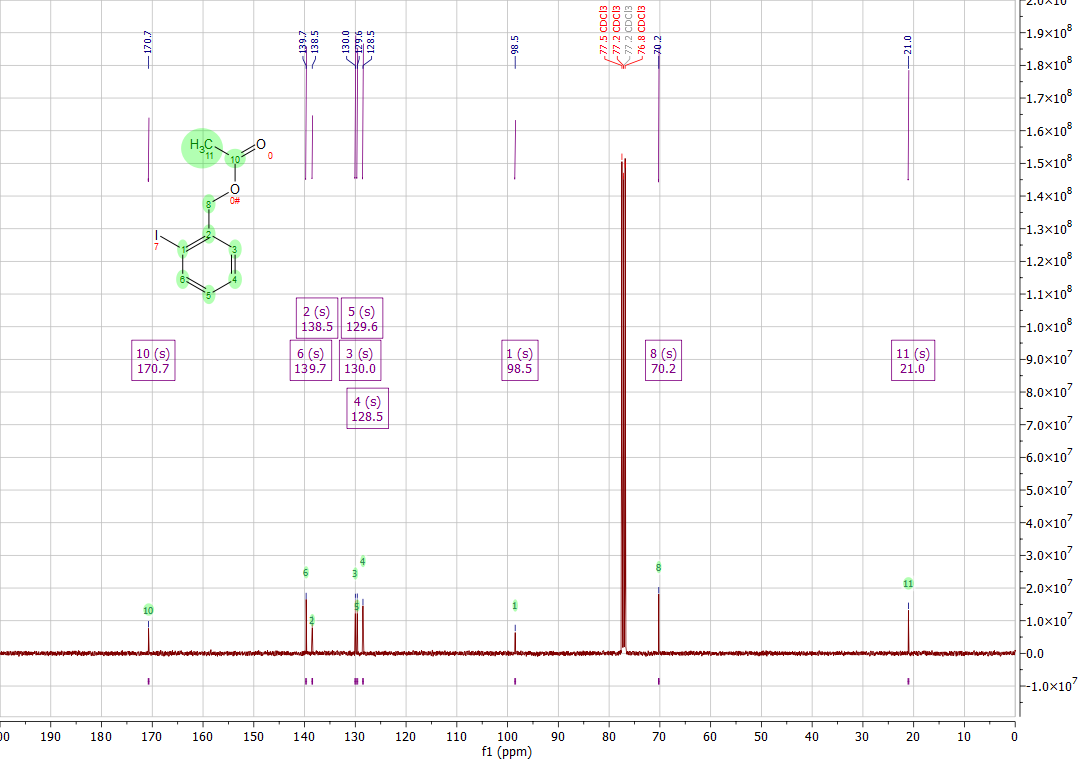


Figure S25. ^13^C NMR spectrum of compound 13

**Acetic acid 3-acetoxymethyl-2-iodo-benzyl ester (14)**


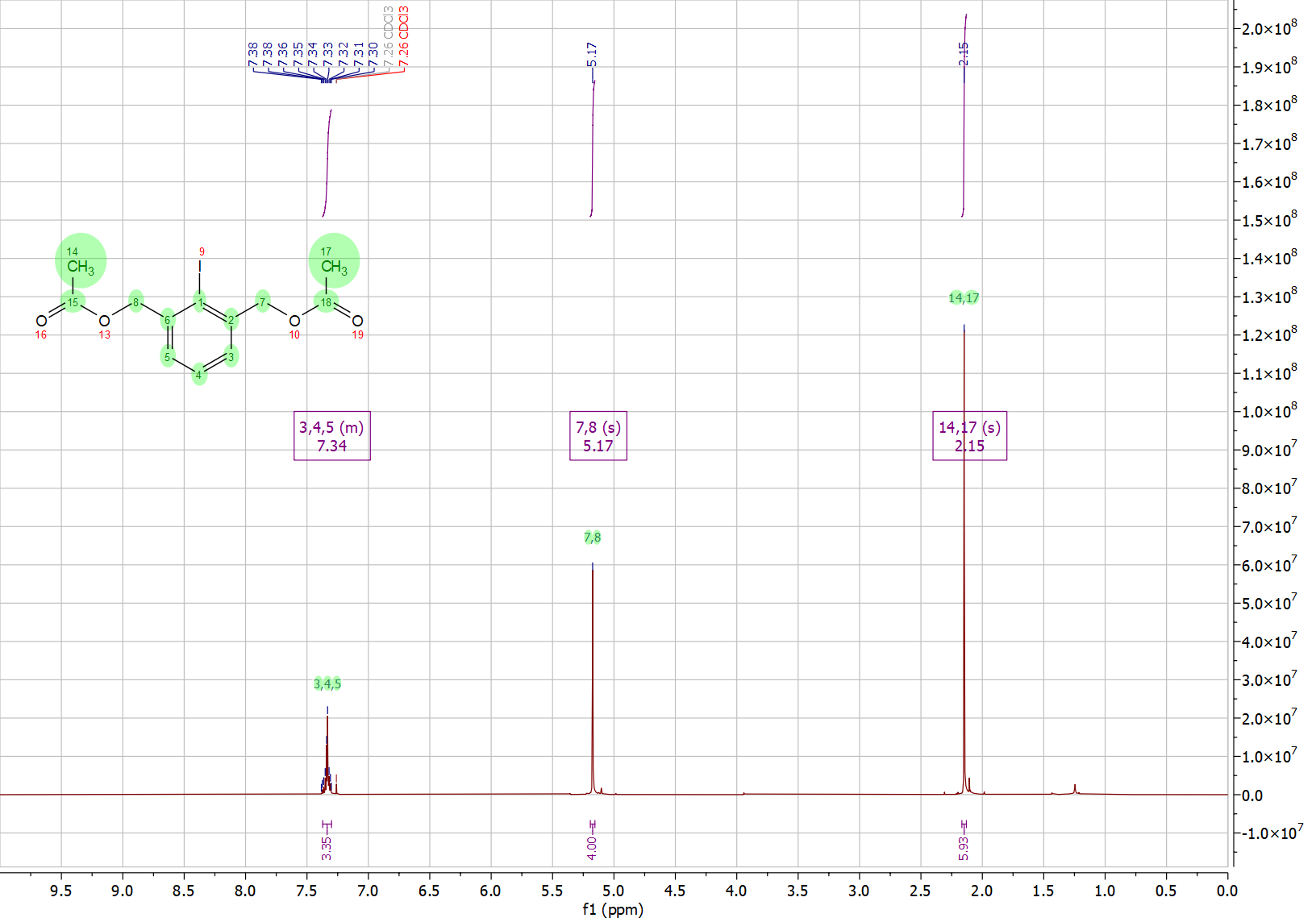


Figure S26. ^1^H NMR spectrum of compound 14


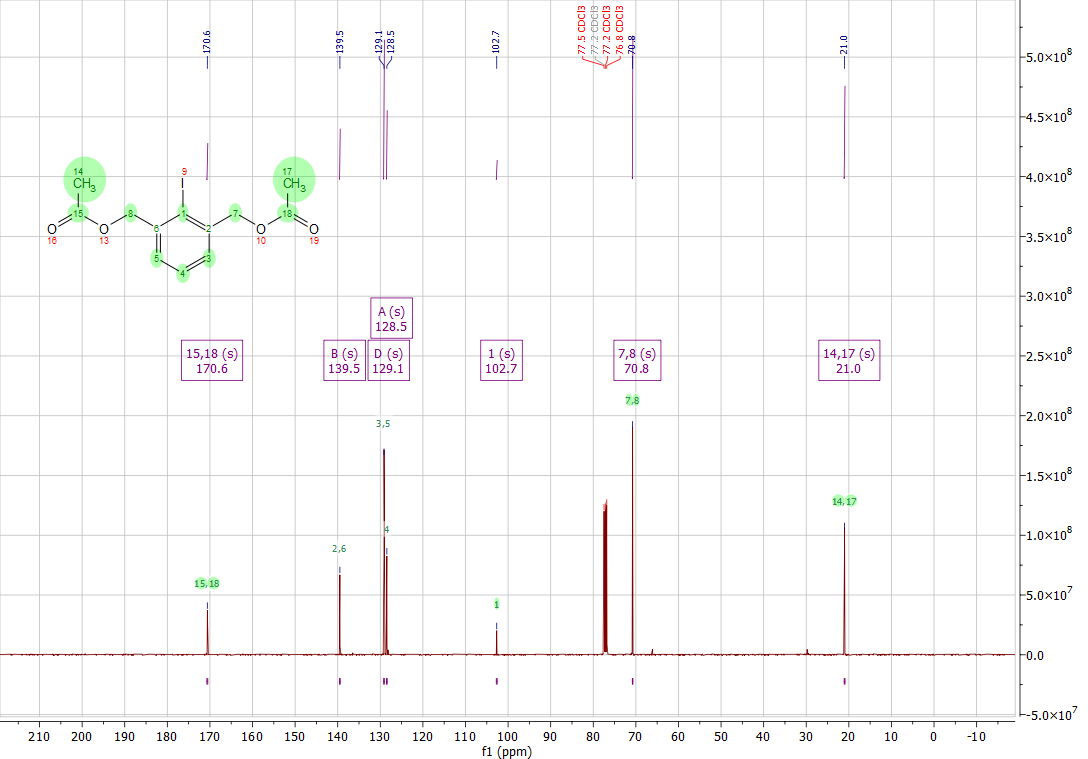


Figure S27. ^13^C NMR spectrum of compound 14

**((2-hydroxymethyl)phenyl)(4-methoxyphenyl)iodonium 4-methylbenzenesulfonate (15)**

**
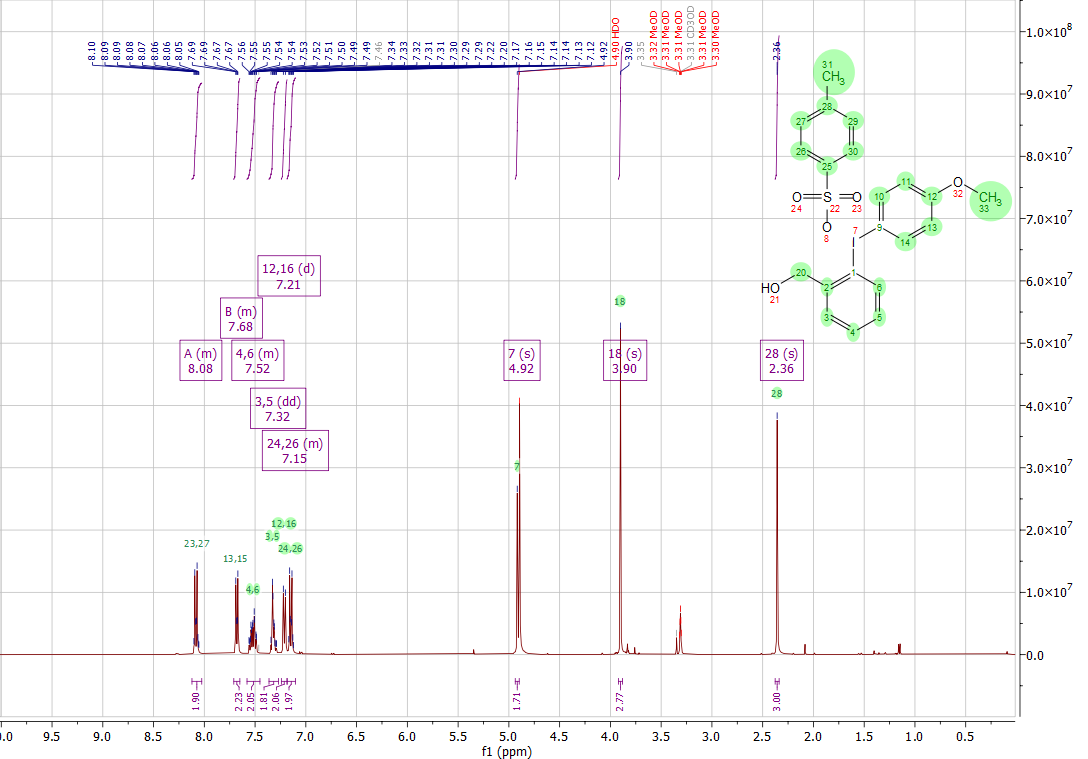
**

Figure S28. ^1^H NMR spectrum of compound 15


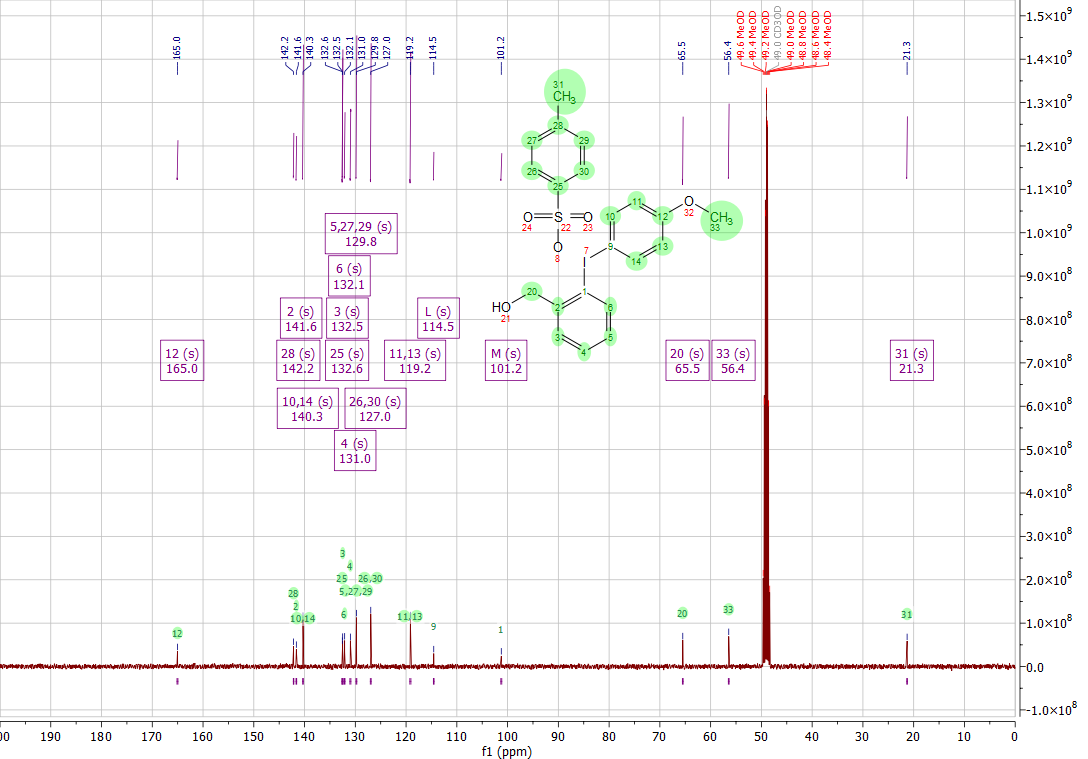


Figure S29. ^13^C NMR spectrum of compound 15


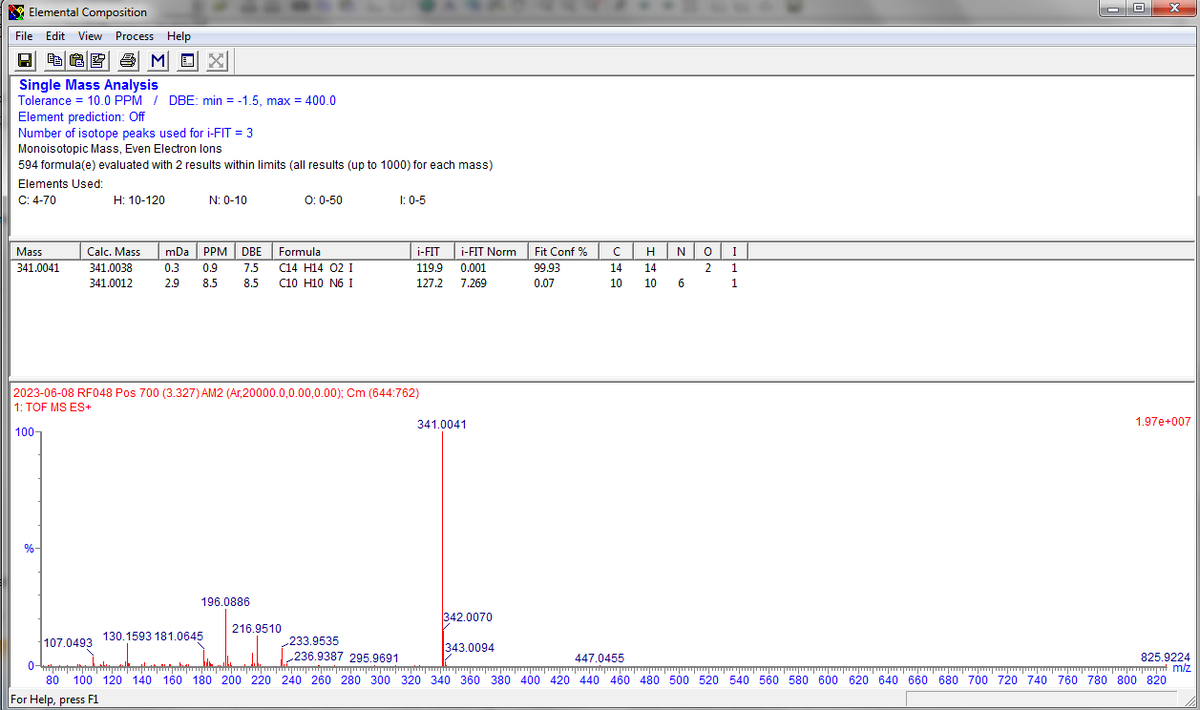


Figure S30. Mass spectrum of compound 15

**(2,6-bis-hydroxymethyl-phenyl)-(4-methoxy-phenyl)-iodonium 4-methylbenzenesulfonate (16)**


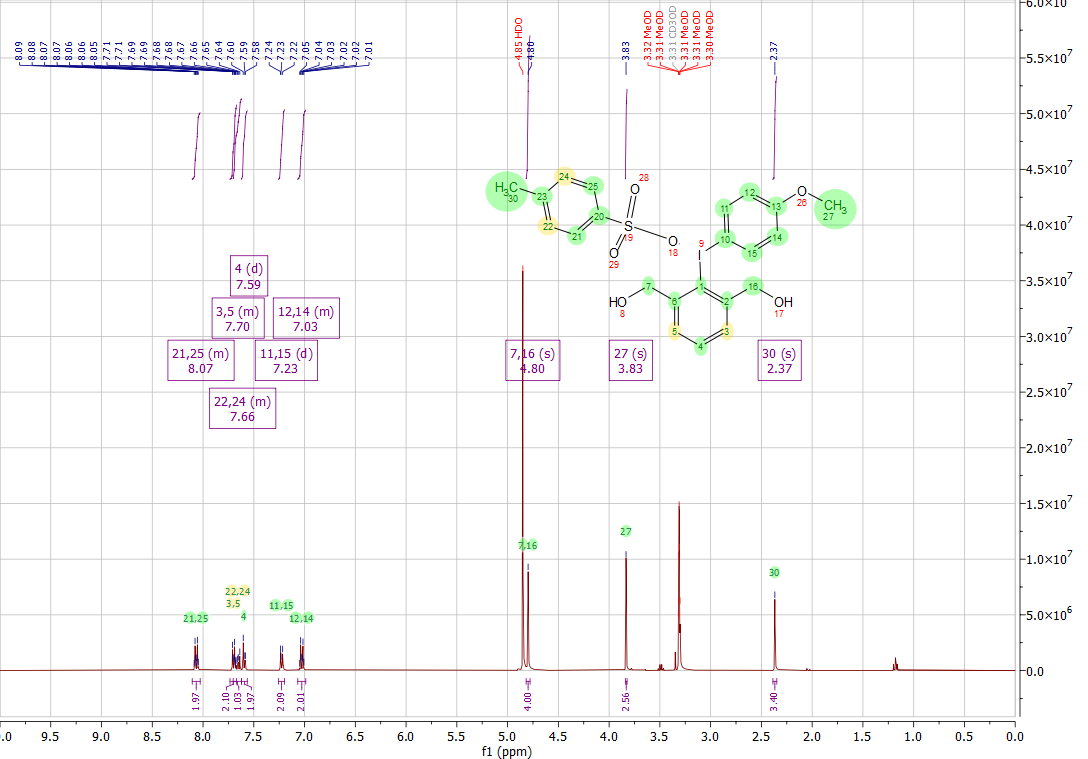


Figure S31. ^1^H NMR spectrum of compound 16


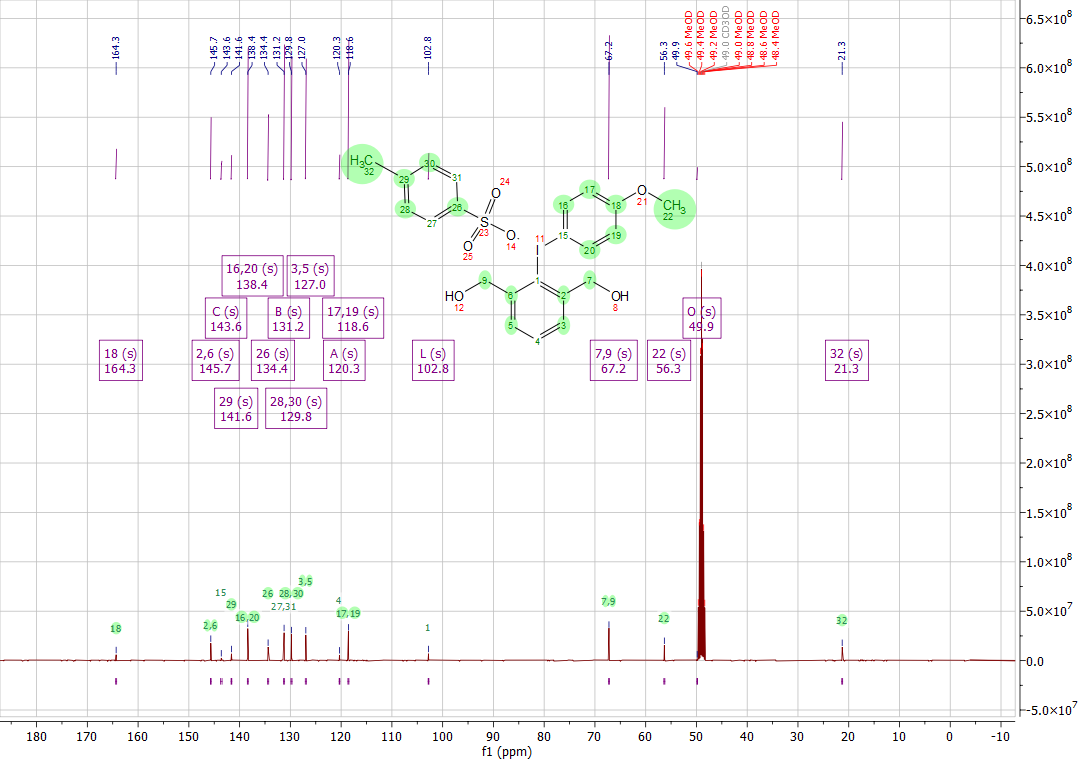


Figure S32. ^13^C NMR spectrum of compound 16


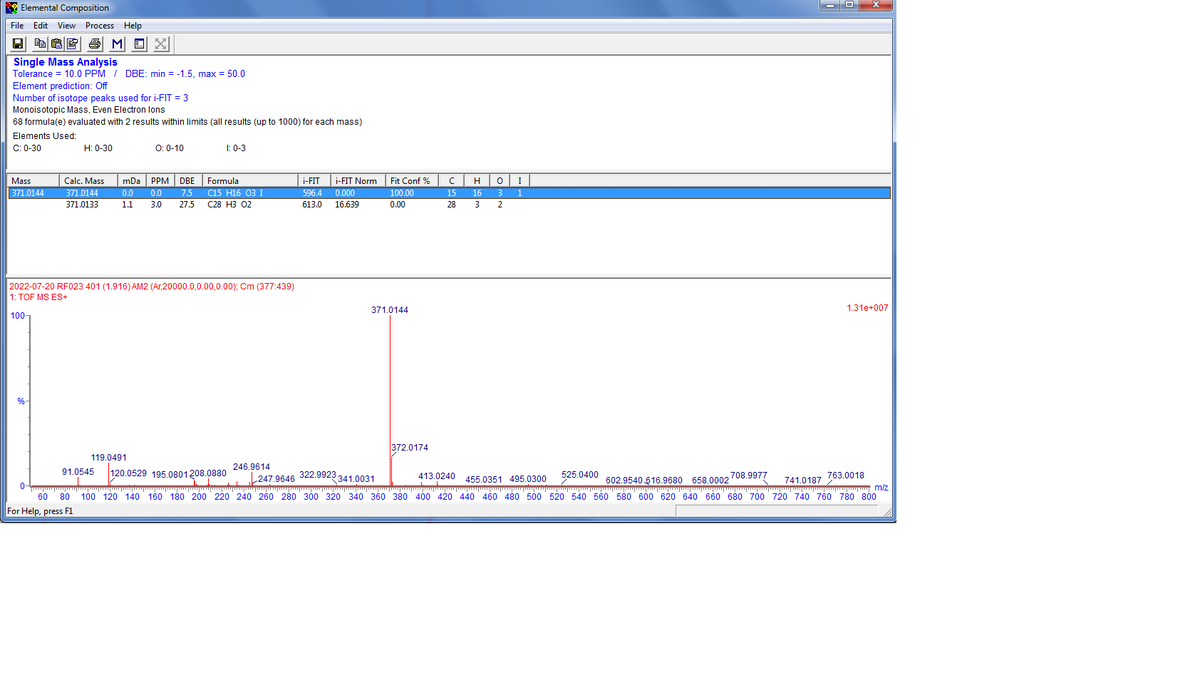


Figure S33. Mass spectrum of compound 16

**3-iodo-*N*-ethylbenzamide (19)**


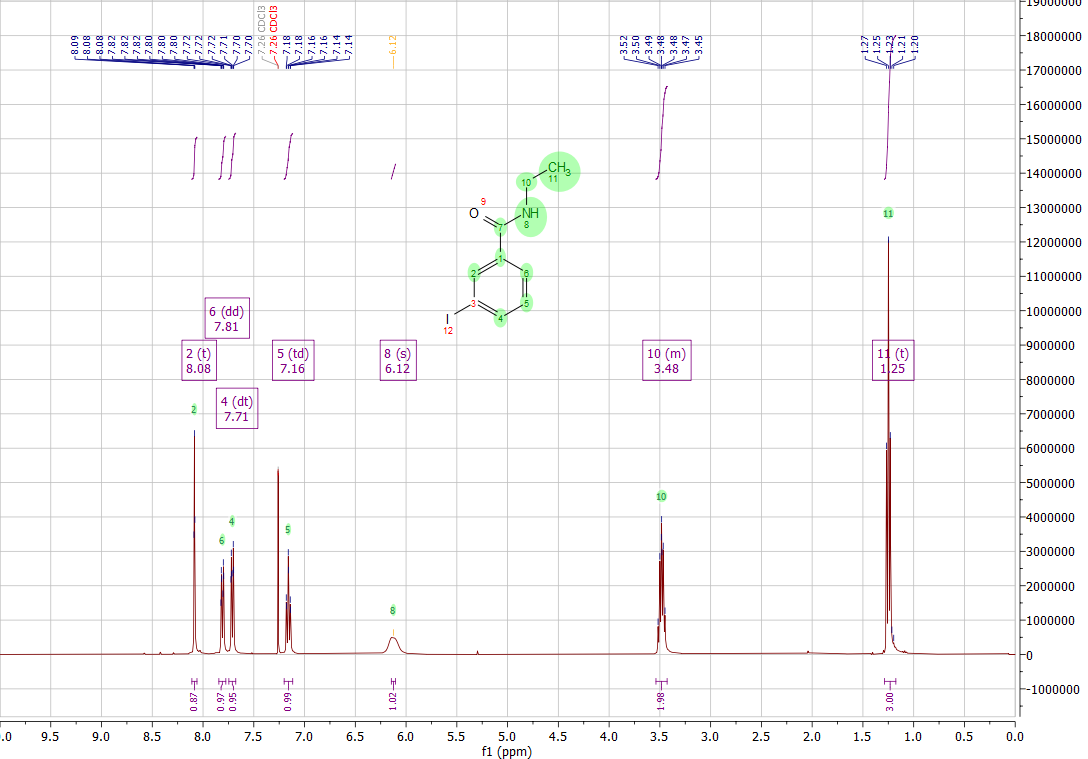


Figure S34. ^1^H NMR spectrum of compound 19


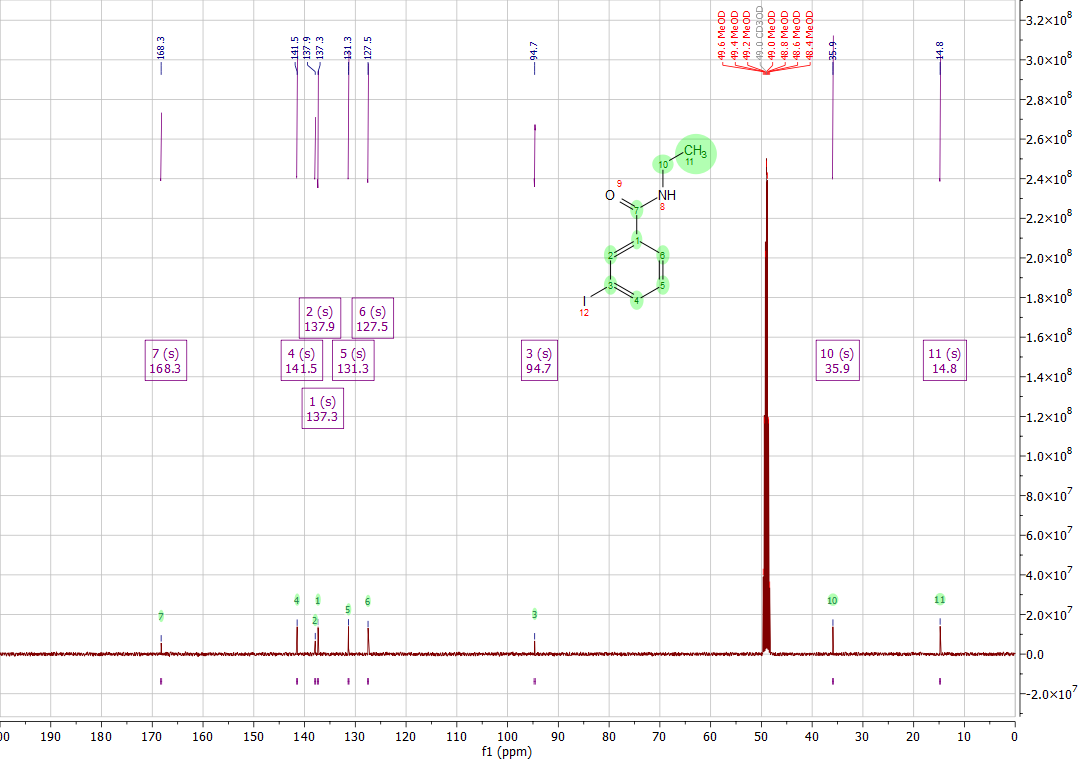


Figure S35. ^13^C NMR spectrum of compound 19

**((3-ethylcarbamoyl)phenyl)(4-methoxy-phenyl)iodonium 4-methylbenzenesulfonate (17)**


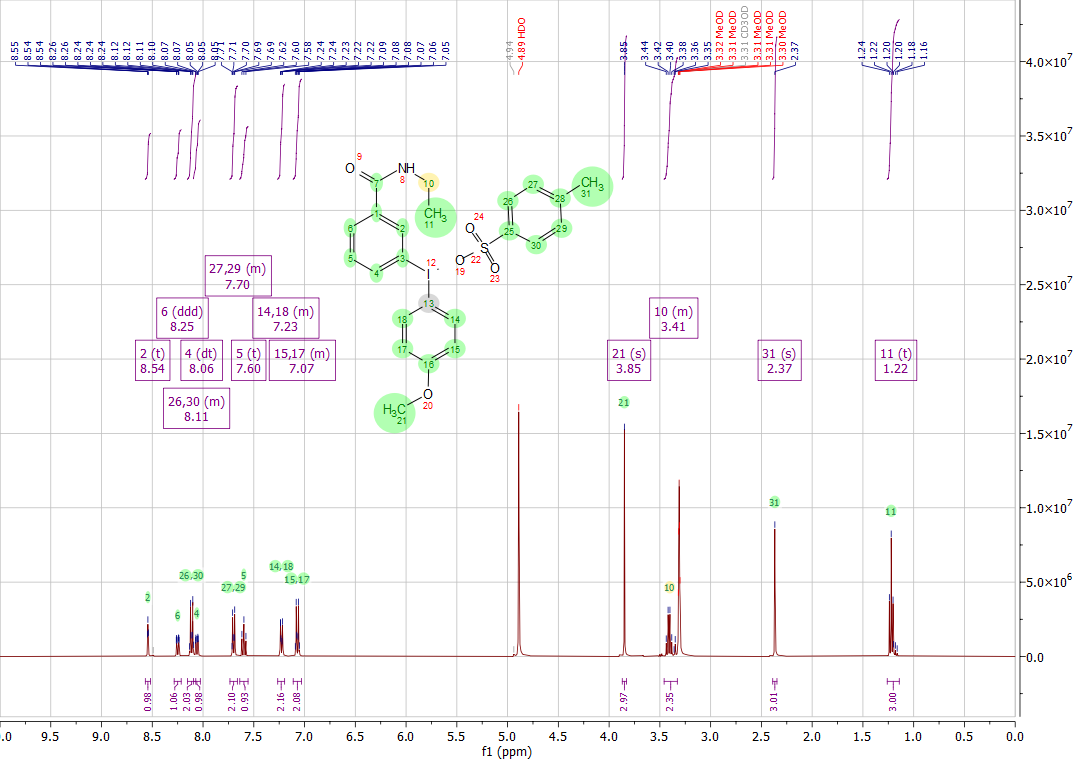


Figure S36. ^1^H NMR spectrum of compound 17


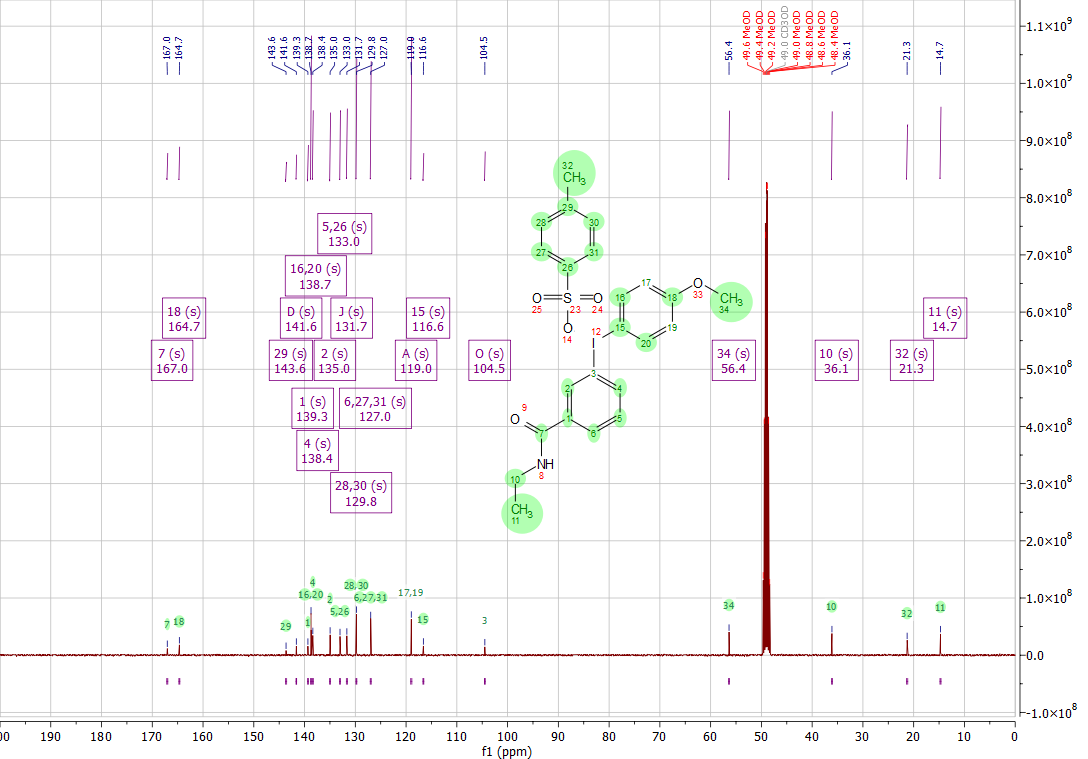


Figure S37. ^13^C NMR spectrum of compound 17


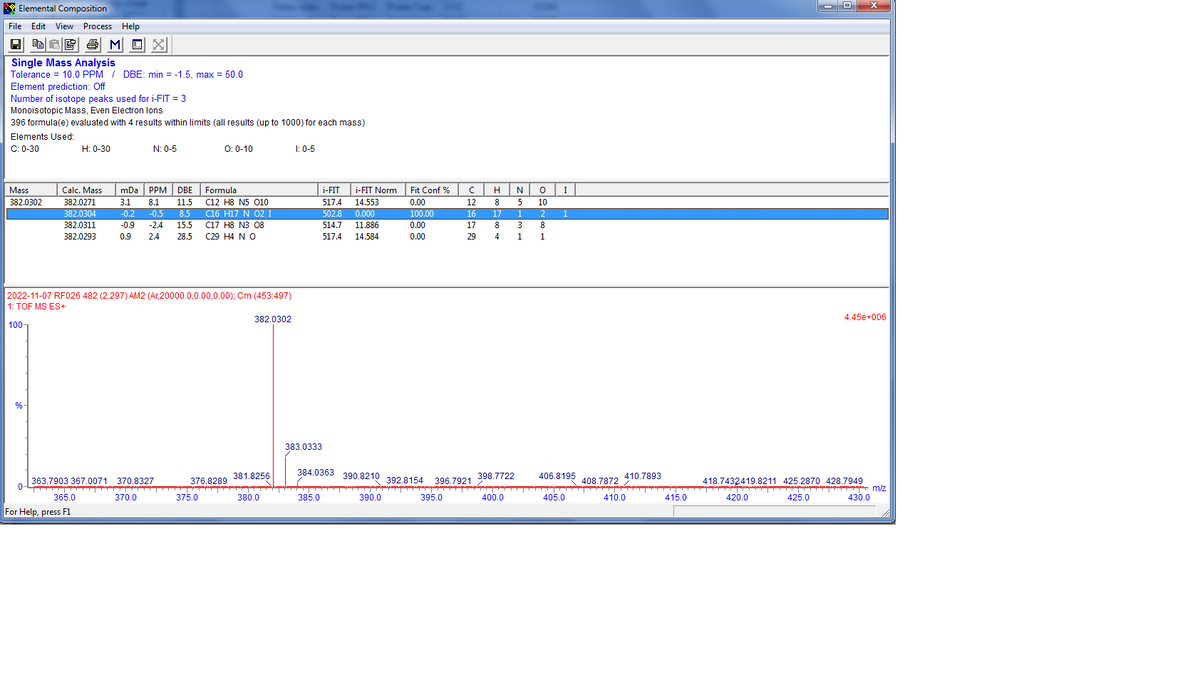


Figure S38. Mass spectrum of compound 17
